# Electromechanical Properties and Structural Regulation of PEDOT-Derived Gels

**DOI:** 10.3390/gels12060502

**Published:** 2026-06-05

**Authors:** Jinjing Cao, Fang Huang, Zhenhao Jiang, Qijin Ge, Zeyu Liu, Zheng Zhao, Feng Chen, Yukun Zhu, Changpo Zhang, Peng Wang, Dongying Wang, Chuizhou Meng

**Affiliations:** 1 School of Information Science and Engineering, Shandong Agriculture and Engineering University, Jinan 250100, China; lancaoer19831230@163.com (J.C.);; 2 School of Mechanical Engineering, University of Jinan, Jinan 250022, China; 3 Department of Materials Science and State Key Laboratory of Molecular Engineering of Polymers, Fudan University, Shanghai 200433, China; 4 Department of Applied Electronics, Shandong Institute of Commerce and Technology, Jinan 250103, China; 5 School of Materials Science & Engineering, Zhejiang Sci-Tech University, Hangzhou 310018, China

**Keywords:** PEDOT, conduction mechanism, material properties, composite synergy

## Abstract

**Poly(3,4-ethylenedioxythiophene)** (PEDOT)-based gels have emerged as a prominent class of functional conductive materials, owing to their unique electromechanical coupling characteristics that integrate electrical functionality and mechanical adaptability. This review systematically elucidates the electromechanical properties of PEDOT-derived gels—defined as the synergistic response of electrical behaviors (conductivity, carrier mobility, electrochemical stability) and mechanical performances (flexibility, stretchability, tensile strength, bending resistance)—under mechanical deformation, as well as their mutual regulatory mechanisms. Focusing on how preparation processes and structural regulation modulate these electromechanical properties, this work first introduces the development history, intrinsic conductive mechanisms, and inherent electromechanical characteristics of PEDOT. It then systematically summarizes mainstream synthesis methods, analyzing their effects on balancing mechanical flexibility and electrical conductivity. Addressing the brittleness and poor electromechanical stability of pure PEDOT, this review further explores composite synergistic mechanisms with conductive/non-conductive polymers, metallic materials, inorganic nanoparticles, and biomaterials, clarifying how interfacial interactions optimize mechanical deformability while preserving or enhancing electrical performance. Finally, it summarizes the applications of PEDOT-based composites in electromechanically compatible fields including flexible sensing, micro/nano patterning, implantable biomedicine, anti-corrosion protection, and energy storage. This review aims to clarify the connotation of PEDOT’s electromechanical properties, refine the focus of relevant research, and provide a theoretical basis for designing high-performance PEDOT-based gels with balanced electromechanical properties.

## 1. Introduction

**Figure 1:**
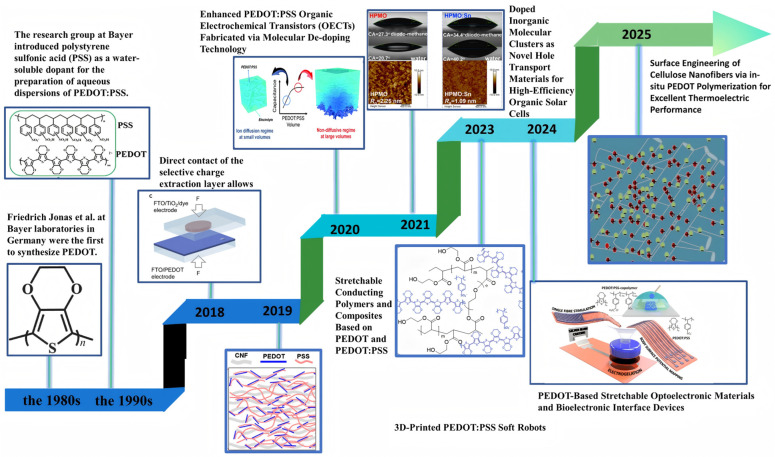
This figure presents the evolution and developmental timeline of PEDOT. This image serves as an illustration of the overall evolution of PEDOT from its emergence to continuous development and applications. The polymerization of PEDOT and the structure of the ionic dopant PSS are shown. The sensitized TiO_2_ electrode and the PEDOT semiconductor-based counter electrode are put in direct contact by mechanically pressing them together to obtain novel DSC embodiment. Reproduced with permission [10], Copyright 2018 Joule. Chemical structure of PEDOT: PSS, its formation of colloidal gel particles when dispersed in water and microstructure of the resulting films with PSS-rich domains (gray) and PEDOT: PSS-rich domains (blue). Reproduced with permission [11]. Copyright 2019 *Nanomaterials*. Effect of amine de-doping on organic electrochemical transistor (OECT) operating voltage and stability. Reproduced with permission [12], Copyright 2020 ACS Omega. Contact angles of the water and diiodo-methane solvent drop on the HPMO-based HTL surface and 2 μm × 2 μm AFM images of the HPMO-based films. Reproduced with permission [13], Copyright 2021 Joule. Roadmap of 3D-printed PEDOT: PSS for soft robotics [14]. Reproduced with permission, Copyright 2023 Macromol Rapid Commun. PEDOT: PSS for stretchable electronics: modifications, processing, and applications. Reproduced with permission [15], Copyright 2024 *Journal of Materials Chemistry C*. The interaction between CNFs and PEDOT. Reproduced with permission [16], Copyright 2025 Sensors and Actuators A: Physical.

The rapid development of flexible electronics and materials has led to significant advances in wearable technologies [1]. Conformal electronics that can wrap around the complex curved surfaces of soft tissues have attracted much attention owing to their innovative application potential in wearable devices, skin electronics, and soft robotics [2]. Poly(3,4-ethylenedioxythiophene) (PEDOT), a leading representative of intrinsically conductive polymers, has become one of the most commercially successful and promising materials in the field of conductive polymers since its initial synthesis by Bayer AG in Germany in 1988. This is due to its excellent conductivity, good environmental stability, tunable redox activity, and unique solution processability [3,4,5]. Since the discovery of polyacetylene in the 1870s, research on conducting polymers has officially commenced. In 1988, Friedrich Jonas and colleagues at Bayer Laboratories in Germany successfully synthesized poly(3,4-ethylenedioxythiophene) (PEDOT) for the first time via chemical/electrochemical oxidative polymerization, achieving a conductivity of approximately 300 S/cm. In 1991, the same research team from Bayer proposed the use of poly(styrenesulfonate) (PSS) as a water-soluble dopant for the preparation of PEDOT: PSS aqueous dispersions. By utilizing PSS as both a template and counterion, they realized the dispersion and film formation of PEDOT in aqueous solutions, endowing the material with integrated superior properties including high conductivity (approximately 10 S/cm), excellent film-forming ability, high visible light transmittance, and remarkable stability. Over the subsequent decades, research and applications of PEDOT continued to expand. This fundamentally addresses the core challenges associated with early-generation conductive polymers (such as polyacetylene, polypyrene, and polythiophene), including poor stability and processing difficulties. In particular, the PEDOT: PSS aqueous dispersion system—formed by blending with polystyrene sulfonate (PSS)—has revolutionarily overcome the processing bottleneck caused by PEDOT’s insolubility and infusibility [6,7,8]. This allows low-temperature solution processing and film fabrication, underpinning large-scale applications in flexible electronics, organic photovoltaics, biosensors and transparent electrodes. Current research on PEDOT-based hydrogels mainly focuses on performance optimization and application verification of single-component systems. Systematic horizontal comparisons of different synthesis and composite strategies, as well as in-depth analysis of research controversies and technical bottlenecks, are still lacking (Figure 1).

This review systematically clarifies the structure–property relationships of PEDOT-based gels, highlights how preparation processes regulate their electrical and mechanical performances, and summarizes their expanding multi-field applications. Starting with the development of PEDOT, it outlines the evolution from EDOT monomer synthesis and polymerization optimization to the commercialization of PEDOT: PSS. By analyzing the molecular structure, it elucidates the fundamental mechanisms underlying PEDOT’s high electrical conductivity, excellent flexibility, and electrochemical stability, thereby providing a theoretical foundation for subsequent research on composite modification [9].

## 2. Mechanism of Electrical Conduction in Electrodes

### 2.1. Overview of PEDOT and PEDOT:PSS

PEDOT is a canonical conjugated conductive polymer belonging to the thiophene family. The ethylenedioxy cyclic structure located at the 3,4-positions of the molecular framework lowers the oxidation potential of monomers and improves the planarity of the polymer main chain, which establishes a continuously delocalized π-electron transport pathway. Benefiting from this structural feature, PEDOT integrates high electrical conductivity, superior visible-light transmittance, excellent thermal-oxidative environmental stability and favorable biocompatibility.

However, pristine PEDOT exhibits rigid molecular chain conformation and poor solubility in water and conventional organic solvents. Its inferior solution processability greatly restricts direct film formation and large-scale practical application. To overcome this critical processing challenge, polystyrene sulfonate (PSS) is widely utilized as both a dopant and dispersant in fundamental research and industrial applications, and aqueous PEDOT: PSS dispersions are fabricated through chemical oxidative polymerization. As a p-type dopant, PSS realizes charge doping of PEDOT to tailor its electrical conductivity; simultaneously, it assembles a core–shell nanomicelle architecture with PEDOT as the core and PSS as the shell, endowing the PEDOT: PSS system with excellent water solubility and desirable processability.

In the literature, the specific parameters of poly(3,4-ethylenedioxythiophene): poly(styrenesulfonate) (PEDOT: PSS) are as follows: a weight ratio of 1:2.5 (with a solid content of approximately 1.1 wt%). For the poly(styrenesulfonate) (PSS) component, the number-average molecular weight (Mn) ranges from 70 to 90 kDa, the weight-average molecular weight (Mw) is between 150 and 200 kDa, and the polydispersity index (PDI) is 2.1–2.5. Secondary doping was conducted using 5 vol% dimethyl sulfoxide (DMSO, Sigma-Aldrich, St. Louis, MO, USA). After magnetic stirring for 30 min, the mixture was spin-coated (spin-coater, Laurell Technologies Corporation, North Wales, PA, USA) into a film at 4000 rpm, followed by annealing (annealing oven, Thermo Fisher Scientific, Waltham, MA, USA) at 120 °C for 15 min.

Furthermore, it is recommended that the spin-coating parameters (4000 rpm, 60 s), ambient humidity (<30% relative humidity, RH), and annealing conditions be reported concurrently. Primarily utilizing PEDOT: PSS with the aforementioned parameters as the main component is critical for improving the reproducibility of results across different laboratories.

### 2.2. Electrical Conduction in Electrodes

As a highly representative intrinsically conductive polymer, poly(3,4-ethylenedioxythiophene) (PEDOT) has become a core electrode material in numerous fields—including flexible electronics, organic photovoltaics, biosensors, and transparent electrodes—thanks to its excellent conductivity, good environmental stability, tunable electrochemical activity, and solution processability [17,18,19,20,21].

#### 2.2.1. Fundamentals of Electrode Structure: Hole-Dominated Transport in Conjugated Structures

Charge transport in conjugated systems essentially stems from the fact that holes formed upon the excitation of π electrons can migrate in an electron-like manner within the conjugated network and the core structural basis of conductivity [22,23,24,25]. It is precisely the synergistic interaction between the conjugated π-electron system and the doped states. Taking PEDOT as an example, its main chain consists of 3,4-ethylenedioxythiophene units linked by conjugated double bonds, forming a delocalized π-electron system that provides the fundamental pathway for charge transport. However, in the undoped state, its electrical conductivity is extremely low (only 10^−8^ S/cm), and conductivity enhancement must be achieved through oxidative doping. During the oxidation process, the molecular chains lose electrons to form positively charged polarons/dipolarons as charge carriers, while anions such as PSS^−^ and CF_3_SO_3_^−^ are introduced to compensate for the lost charge and maintain electrical neutrality. The degree of delocalization of the conjugated π electrons directly determines the fundamental efficiency of charge transport, and the ethylene dioxide substituents on PEDOT stabilize the conjugated structure and enhance carrier mobility, Furthermore, the type of dopant (e.g., PSS^−^, Tos^−^) significantly influences carrier concentration and interchain interactions; for instance, PSS^−^ improves solubility, while small-molecule anions are more effective in enhancing conductivity [26].

#### 2.2.2. Polaron Migration and Ion-Assisted Charge Compensation

Charge compensation refers to the phenomenon in which, during crystal doping, when charged centers are formed due to unequal substitution, opposite charges are introduced to maintain electrical neutrality. During crystal doping, unequal substitution creates charged centers within the crystal. To preserve crystal’s electrical neutrality, a center with an opposite charge must be introduced; this process is known as charge compensation.

Oxidative doping causes PEDOT chains to form monopoles (single-electron deficiency) or dipoles (double-electron deficiency); these quasiparticles couple with structural distortions in the chains and, under an applied electric field, migrate along the conjugated main chain or via interchain hopping, generating a hole current [26]. Charge transport is categorized into ultrafast intra-chain transport, medium-rate inter-chain hopping, and slow inter-chain transport. Among these, the π–π stacking distance is the rate-limiting step for inter-chain transport; shortening the stacking distance can significantly enhance conductivity [26,27,28]. At the electrode–electrolyte interface, the migration of anions or electrolyte ions (such as Na^+^ and H_3_O^+^) can aid in charge compensation; particularly in electrochemical applications, charge transfer is achieved through the synergistic transport of ions and electrons/holes. Ion transport in PEDOT:PSS occurs primarily through nanoscale channels and the sulfonic acid groups of PSS, while electron transport is concentrated within the PEDOT matrix; the efficiency of combined transport is regulated by the microstructure [29]. Currently, there remain two major controversies and limitations in the research on the conductive mechanism of PEDOT: ① No consensus has been reached on the primary–secondary relationship between polaron migration and ion-assisted conduction: some studies argue that interchain polaron hopping is the core mechanism, while others emphasize the dominant role of electrolyte ion diffusion, with the discrepancy stemming from differences in test environments (dry/wet states). ② Existing models overlook the influence of microscale defects: practical PEDOT gels exhibit chain entanglement and defect sites, and current theories struggle to explain the fluctuations in conductive performance induced by these defects. ③ Lack of in situ characterization support: most mechanistic conclusions are based on static tests, which fail to reflect the real-time changes in charge transport during dynamic charge–discharge/deformation processes.

### 2.3. Formation Mechanism of the Electrical Properties of PEDOT

#### 2.3.1. Conductivity (σ)

Conductivity is a physical parameter characterizing the ability of a material to conduct electric current, which is used to measure the difficulty of current conduction in the material. Pure PEDOT exhibits a relatively low intrinsic conductivity of approximately 7.5 S/m (equivalent to 0.0075 S/cm). Specifically, the conductivity of conventional PEDOT: PSS ranges from 10^−3^ to 10^3^ S/cm. The π–π stacking distance acts as the rate-limiting factor governing interchain charge transport and exhibits a strongly negative correlation with electrical conductivity. Within the range of 3.43–3.70 Å, every 0.05 Å reduction in stacking distance can elevate conductivity by 2–5 times. For instance, reducing the π–π stacking distance from 3.50 Å to 3.43 Å enables the conductivity of PEDOT films to surge from 660 S/cm to 7520 S/cm, delivering an enhancement of up to 1140%. Similarly, post-solvent treatment of PEDOT: PSS diminishes the stacking distance from 3.53 Å to 3.48 Å, accompanied by a remarkable conductivity increase from 120 S/cm to 3360 S/cm. Such exceptional performance improvement stems from the strengthened interchain overlap of π-orbitals and facilitated polaron/bipolaron hopping, which fundamentally circumvents the intrinsic bottleneck of interchain charge transport. Post-treatment methods, such as the sulfonation degree of dopants, solvent type, acid treatment, and thermal annealing, can regulate the molecular chain packing mode, crystallinity, and orientation of PEDOT, improve the interchain π–π stacking degree, optimize the connectivity of the conductive network, and thereby synergistically enhance the conductivity. For high-performance PEDOT: PSS modified by secondary doping such as DMSO treatment or acid treatment, the conductivity can reach 4000~6259 S/cm, with a maximum value of up to 1.06 × 10^4^ S/cm. For self-doped PEDOT without any additives, the maximum conductivity can also reach 1089 S/cm.

#### 2.3.2. Carrier Mobility

**Figure 2:**
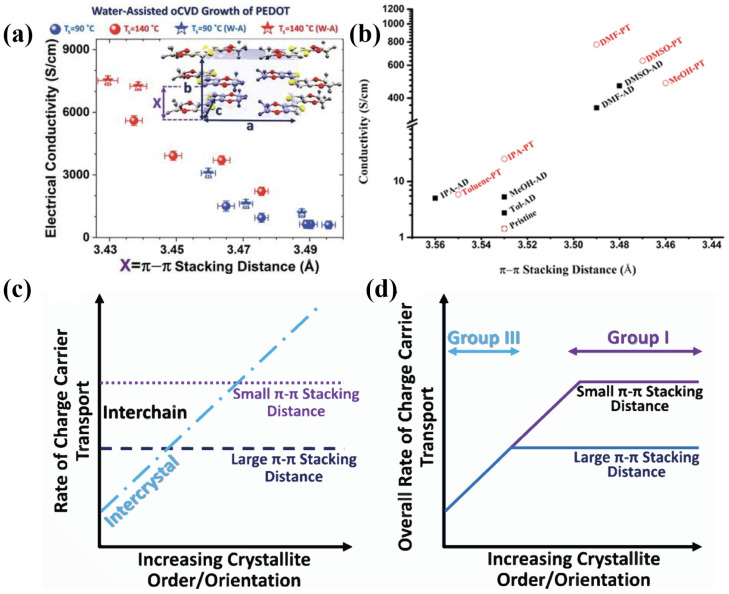
This figure illustrates the influence of π–π stacking on the electrical conductivity of PEDOT thin films, with a focus on the relationship between the π–π stacking distance and electrical conductivity. (**a**) The relationship between the electrical conductivity of PEDOT thin films and the π–π stacking distance [30]. (**b**) Film conductivity against the main key structural parameters: (**a**) π–π stacking distance [31]. (**c**) The inter-chain transport rate for two different π–π stacking distances and the inter-crystallite transport rate. The inter-chain transport rate changes as more linear tie chains form with an increase in the order and orientation in conjugated polymers [32]. (**d**) The overall rate of charge transport in conjugated polymers.

Carrier mobility (μ, unit: cm^2^ V^−1^ s^−1^) describes carrier transport speed under an electric field, directly determining material conductivity and device response speed. For PEDOT, μ depends on π-electron delocalization continuity, molecular chain conformation, film phase separation, and stacking order: conjugated orbital overlap ensures intrachain transport, while sulfonation degree, solvent, and regular π–π stacking regulate film morphology and interchain transport (Figure 2).

PEDOT exhibits typical p-type semiconductor characteristics with dominant hole mobility. Conventional PEDOT: PSS has a μ of 1~10 cm^2^ V^−1^ s^−1^, high-performance oCVD-prepared PEDOT films reach up to 18.45 cm^2^ V^−1^ s^−1^, and ionic mobility in PEDOT: PSS is ~2.2 × 10^−3^ cm^2^ V^−1^ s^−1^. Carrier transport relies on hopping and band conduction in the conjugated π-system, affected by intermolecular interactions and crystal structure.

#### 2.3.3. Carrier Concentration

Carrier concentration refers to the number of charge carriers (such as electrons and holes) involved in conduction per unit volume of the material, which is one of the core parameters determining the electrical conductivity of the material, with the common unit of cm^−3^. A higher carrier concentration means more charge particles available for conduction in the material. The carriers are mainly polarons/bipolarons generated by oxidative doping, and their concentration is jointly determined by the generation and retention of carriers. Undoped PEDOT has almost no effective carriers, resulting in an extremely low carrier concentration (n). The higher the intensity of chemical/electrochemical oxidative doping, the more electrons lost by the molecular chain, and the greater the number of positive polarons and bipolarons generated, leading to a significant increase in n. The type and effect of counterions: counterions such as PSS^−^ and Cl^−^ are embedded into the system to compensate for the positive charge of the molecular chain, maintain electrical neutrality, inhibit carrier charge recombination, reduce carrier loss, and effectively retain and improve n. The typical carrier concentration of PEDOT at room temperature is 10^21^~10^22^ cm^−3^, and the carrier concentration of highly conductive PEDOT films can reach 8.66 × 10^22^ cm^−3^. The carrier concentration and conductivity satisfy the relationship σ = neμ, where it is the electronic charge. The carrier concentration of PEDOT can be effectively adjusted by regulating the doping level, thereby modulating its electrical conductivity.

### 2.4. Mechanical Properties of PEDOT

#### 2.4.1. Elastic Modulus (Young’s Modulus)

The elastic modulus characterizes the ability of a material to resist elastic deformation. The modulus of PEDOT varies significantly depending on its morphology and preparation conditions: Pure PEDOT: PSS hydrogel is 2–10 MPa, which is equivalent to soft elastomers such as PDMS, making it suitable for the mechanical environment of biological tissues. Dense PEDOT: PSS film is 1–5 GPa, which is greatly affected by humidity (hydrogen bonds weaken when the humidity is higher than 40%, leading to a decrease in modulus). Composite modified system: Adding flexible components such as PEO and natural rubber can reduce the modulus to 100 kPa–1 MPa, improving flexibility and compliance.

#### 2.4.2. Tensile Strength and Elongation at Break

Tensile properties determine the load-bearing and deformation capacity of a material under stress, and the tensile behavior of PEDOT can be significantly regulated through modification. The tensile strength of pure PEDOT: PSS films is 10–50 MPa, with an elongation at break of only 5~15%, indicating high brittleness and limited tensile deformation capacity. For secondary doped PEDOT: PSS treated with DMSO or acid, the tensile strength is 8–40 MPa, and the elongation at break is increased to 15~60%, showing significantly improved ductility. The tensile strength of PEDOT: PSS and elastomer composite materials is 1–10 MPa, with an elongation at break of up to 300~500%, among which the elongation of the composite system with natural rubber can reach up to 490%. Self-doped PEDOT such as PEDOT: OTf has a tensile strength of 5–20 MPa and an elongation at break of 20~30%, possessing intrinsic stretchability without the need for elastomer modification.

#### 2.4.3. Flexibility and Bending Performance

Flexibility is the most prominent mechanical advantage of PEDOT, which is far superior to traditional inorganic conductive materials (e.g., ITO): Bending limit: It can withstand repeated bending with a radius of curvature as low as 0.1 mm, while ITO usually fractures when the radius of curvature is greater than 5 mm. Bending stability: The resistance change is less than 5% after 100 bending cycles; the AgNWs-PEDOT:PSS composite electrode still has a resistance change of less than 0.2 after 50,000 U-shaped folds. Adaptability: It can fit complex curved surfaces, making it suitable for application scenarios such as wearable devices and flexible touch screens (Table 1).

## 3. PEDOT Synthesis Process

### 3.1. Solution-Phase Synthesis

The solution-phase synthesis method is the most traditional and widely used approach for preparing PEDOT, and its core lies in the oxidation–polymerization reaction [33,34]. Due to its electron-rich thiophene ring structure, the EDOT monomer loses an electron under the action of oxidizing agents (such as FeCl_3_, Na_2_S_2_O_8_, or Fe (III) Tos) to form a cationic radical. Two radicals couple and lose a proton, forming a dimer, which then continues to oxidize and couple, forming a long polymer chain. During the polymerization process, the PEDOT main chain carries a positive charge due to its conjugated structure. It relies on anions in the solution (such as PSS^−^, Cl^−^, and SO_4_^2−^) to function as dopants that enter the polymer backbone to balance the charge, thereby imparting good electrical conductivity.

#### 3.1.1. Chemical Oxidative Polymerization

EDOT monomer is added dropwise to a solution containing an oxidizing agent (such as FeCl_3_) and a dopant (such as camphor sulfonic acid or p-toluenesulfonic acid), and the reaction is conducted under stirring conditions. The polymerization product is an insoluble, infusible, deep blue PEDOT powder that requires further processing (such as dispersion and film formation) before it can be used in devices. This method is simple to operate and suitable for batch synthesis, but the product morphology is difficult to control, and its conductivity is low. Studies have shown that the polymerization reaction rate is determined by the step in which the EDOT monomer is oxidized by the oxidizing agent to generate free radicals; this rate-limiting step governs the overall polymerization kinetics.

#### 3.1.2. Emulsion Polymerization

EDOT monomers are added to an aqueous solution of polystyrene sulfonate (PSS) in excess to form a micellar or emulsion system. PSS plays three key roles in this process: as a surfactant to disperse the monomer, as a dopant to balance the positive charge of PEDOT, and as a polymeric scaffold to guide the growth of PEDOT chains on its surface, thereby forming stable PEDOT: PSS composite particles. The introduction of PSS has revolutionarily solved the processing challenges associated with PEDOT, enabling the commercial production of PEDOT:PSS aqueous dispersions [35]. Since its first commercial application in 1990 as an antistatic coating for Agfa film (Agfa-Gevaert N.V., Mortsel, Antwerp, Belgium), PEDOT:PSS has evolved into several commercial product lines, including Clevios™ and Orgacon^®^ [35].

#### 3.1.3. Emerging Liquid-Phase Polymerization Methods

Liquid-phase polymerization (LPP) is an improved liquid-phase polymerization method. First, an oxidant solution (such as ammonium metavanadate or Fe (III)Tos) is spin-coated or spray-coated onto the substrate, followed by the application of an EDOT monomer solution. This causes the polymerization reaction to occur at the liquid–liquid interface on the substrate surface, directly forming a uniform PEDOT film. Furthermore, to overcome the acidity and hygroscopicity issues associated with PSS (which may reduce device lifetime and performance), researchers have developed organic solvent-dispersed systems based on block copolymers. For example, by forming a block copolymer with flexible, soluble polymers such as polyethylene glycol (PEG), the PEDOT blocks are stabilized by the steric hindrance of the PEG chains, allowing for the formation of stable colloidal dispersions in polar, non-protonic solvents such as propylene carbonate or nitromethane. By controlling the block composition, molecular weight, block ratio, and type of dopant, the bulk electrical conductivity of the copolymer can be adjusted within the range of 10^−4^ S/cm to 60 S/cm. Solution synthesis, particularly emulsion polymerization, stands as the mainstream technology for the industrialization of poly(3,4-ethylenedioxythiophene): poly(styrenesulfonate) (PEDOT: PSS). This approach boasts prominent advantages including straightforward operation, low cost, and scalability for large-scale fabrication. However, it suffers from poor reaction controllability, which leads to uneven chain lengths of PEDOT and inhomogeneous distribution of PSS. Consequently, the electrical conductivity of products fluctuates significantly across different batches, ranging from 10^−4^ to 60 S/cm. Notably, there exist substantial discrepancies in the performance reported by different research groups. The core underlying reason lies in the lack of a unified standard for the PSS doping ratio and oxidant dosage. Furthermore, the post-treatment process of the products is cumbersome, and residual impurities inevitably compromise the long-term stability of the final materials.

### 3.2. Vapor-Phase/Interfacial Deposition Method

The vapor-phase/interface deposition method enables the in-situ growth of PEDOT films by confining the polymerization reaction to the gas–solid or liquid–solid interface. The core of this method lies in pre-fixing the oxidizing agent on the substrate surface; EDOT, present in the vapor phase, is adsorbed onto the substrate and undergoes polymerization upon contact with the oxidizing agent.

#### 3.2.1. Vapor-Phase Polymerization (VPP)

An oxidizing agent (such as Iron p-toluenesulfonate, Fe (III)Tos) is dissolved in a solvent and then spin-coated or spray-coated onto a substrate to form a thin oxidizing agent layer. The substrate is then placed in a sealed chamber filled with EDOT vapor; EDOT molecules are adsorbed onto the surface of the oxidizing agent layer and undergo polymerization, forming a highly conductive, structurally dense PEDOT film. In 2004, Wanke Cheng introduced [36] pyridine as a basic inhibitor, which not only slowed the polymerization rate but also eliminated acidic side reactions, resulting in PEDOT films with a conductivity exceeding 1000 S/cm. Oxidative chemical vapor deposition (oCVD) is a further development of VPP, in which the oxidant and monomer meet on the substrate surface in vapor form and undergo oxidative polymerization, thereby avoiding the deposition of the oxidant on the substrate and making it suitable for highly volatile oxidants.

#### 3.2.2. Low-Melting-Point Solvent-Induced Vapor-Phase Polymerization

This is a sophisticated patterning technique [36]. The researchers designed a deep eutectic solvent (DES) composed of an oxidizing agent (FeCl_3_·6H_2_O) and hydrogen-bonding donors (such as urea and acetamide) to serve as a “reactive ink.” This DES system not only provides the oxidizing agent, but its components (such as urea) can also form dynamic hydrogen bonds with the substrate (such as hydroxyl-rich cellulose) and the EDOT monomer. Urea acts as a “molecular guide,” effectively trapping and immobilizing the EDOT monomer, ensuring that polymerization occurs only in the areas coated with the DES [36]. By “drawing” DES onto the substrate in advance using direct writing or printing techniques, it is possible to achieve micron-scale patterned polymerization of PEDOT on substrates such as paper, wood, and plastic [36]. This method achieves a patterning resolution of 200–400 μm, and the resulting PEDOT/cellulose material exhibits high electrical conductivity (282 S/m), high specific surface area (5.29 m^2^/g), and high areal capacitance (2220 mF/cm^2^) [36]. In a stirred DES-monomer system, a liquid–liquid micro-interface also forms. Polymerization occurs at the interface between EDOT monomer microdroplets and the DES; the resulting PEDOT chains diffuse into the DES phase and self-assemble under the influence of the amide molecules, forming a unique porous structure [36]. Vapor phase polymerization (VPP) enables the fabrication of highly conductive PEDOT films with a conductivity exceeding 1000 S/cm, featuring remarkable advantages of uniform film morphology and high crystallinity. However, this approach is hindered by high equipment costs and harsh reaction conditions (requiring a sealed high-temperature chamber), which renders large-area fabrication challenging. Although deep eutectic solvent (DES)-induced deposition has achieved micropatterning, its upper resolution limit (200–400 μm) is substantially lower than that of photolithography technology. Furthermore, it exhibits narrow substrate compatibility, being only applicable to soft materials such as paper and cellulose, thus limiting its practical application scenarios. Liquid-phase synthesis features mature, low-cost, and scalable fabrication of aqueous PEDOT: PSS dispersions with excellent film-forming capability, superior flexibility, and facile solution processability, yet it suffers from relatively low electrical conductivity, moderate film uniformity, and inferior electrochemical and structural stability. By contrast, vapor-phase polymerization (VPP) and oxidative chemical vapor deposition (oCVD) enable the synthesis of highly crystalline, electrically conductive, uniformly dense pure PEDOT films with negligible solvent residues, exceptional stability, and conformal coverage on complex substrates, albeit at the expense of sophisticated instrumentation, stringent reaction conditions, elevated costs, and challenges in large-area manufacturing.

Beyond electrochemical performance and processing precision, environmental footprint has emerged as an increasingly critical screening criterion for evaluating the practicality of PEDOT fabrication methods. Notably, vapor-phase polymerization (VPP) and water-borne emulsion polymerization (the industrial route for PEDOT: PSS) exhibit substantial differences in environmental impact, warranting in-depth discussion.

Environmental Burden of Vapor-Phase Polymerization (VPP): VPP typically employs volatile organic compounds (VOCs) to dissolve oxidants (e.g., Fe (III) tosylate in butanol or methanol), with reactions assisted by vacuum or inert gas flow. Unreacted EDOT monomers and solvent volatiles must be collected via cold traps or scrubbers to avoid direct emission. Common solvents (e.g., methanol, isopropanol, acetonitrile) are toxic or hazardous VOCs, whose production, transportation, and post-treatment increase the carbon footprint. Additionally, VPP operates at relatively high temperatures (60–120 °C) under vacuum, leading to high energy consumption. Despite yielding ultra-high conductivity (>1000 S/cm) and uniform thin films, VPP’s high environmental cost renders it unsuitable for green electronics manufacturing with stringent sustainability demands.

**Figure 3:**
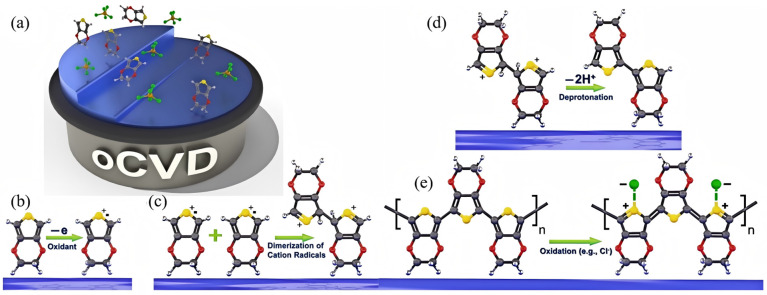
Schematic of step-growth polymerization in oxidative chemical vapor deposition (oCVD): (**a**) Vapors of monomer (EDOT) and oxidant (SbCl_5_) are delivered onto a temperature-controlled substrate. (**b**) EDOT monomers are oxidized to form radical cations. (**c**) Radical–radical coupling yields dimers. (**d**) Dimer deprotonation establishes conjugated structures. (**e**) Counterion dopants incorporate into the PEDOT backbone [37].

Environmental Friendliness of Emulsion Polymerization (Solution-Based Method): In contrast, emulsion polymerization for industrial PEDOT: PSS production uses water as the continuous phase, with polystyrenesulfonic acid (PSS) serving as both template and dopant, eliminating the need for additional organic solvents. Reactions proceed at atmospheric pressure and room or moderately elevated temperatures (<80 °C), resulting in low energy consumption. The system is dominated by water, with only minor by-products (inorganic salts and low-concentration unreacted monomers) that can be degraded via conventional wastewater treatment. Furthermore, aqueous dispersions are directly applicable to processes such as spin-coating and inkjet printing, obviating organic solvent post-treatment. While its conductivity (10^−3^–10^3^ S/cm) and crystallinity are generally lower than those of pure PEDOT prepared by VPP, it offers irreplaceable advantages in environmental friendliness, scalability, and biocompatibility (Figure 3).

Trade-Offs and Recommendations: Material selection thus requires a balance between performance and environmental sustainability. For high-performance devices (e.g., high-frequency organic electrochemical transistors, transparent electrodes), the VPP route remains appealing but should adopt low-toxic solvents (e.g., ethyl lactate instead of methanol) and closed-loop recovery systems. For wearable electronics, biosensors, and large-area flexible substrates, aqueous emulsion polymerization should be the preferred route. Future research should systematically quantify life cycle assessment (LCA) indicators for different synthesis routes—including energy consumption, VOC emission equivalents, and water consumption per unit area of thin film—to establish a more comprehensive material screening framework.

### 3.3. Electrochemical Polymerization

Anodic oxidation polymerization is an electrochemical route to deposit conductive polymers within a three-electrode cell. The working electrode (e.g., ITO or Au) is immersed in an EDOT-containing electrolyte. Upon applying an oxidation potential, EDOT is oxidized to cationic radicals, followed by coupling and polymerization to form PEDOT films directly on the electrode surface. Polymerization and doping proceed simultaneously, with anions (e.g., ClO_4_^−^, fullerene anions, PSS^−^) incorporated into the PEDOT matrix. Film thickness, morphology, and redox states can be precisely tuned by current density, CV cycles, and potential profiles. followed by electrochemical polymerization to introduce functional groups into the PEDOT backbone, thereby preparing functionalized PEDOT films [38,39]. Electrochemical polymerization offers precise control over film architecture and one-step fabrication, yet it is inherently limited by substrate dependence, small deposition area, high cost, and poor scalability. Moreover, electrolyte variations lead to inconsistent electrochemical stability data, impeding standardized protocols.

### 3.4. Radiation Polymerization

Radiation polymerization relies on high-energy irradiation (γ-rays or electron beams) to generate reactive species (e_a_q^−^, •OH, H•) from solvents, which initiate EDOT polymerization. Two pathways exist: oxidative irradiation in N_2_O-solution produces hydroxyl radicals for cationic polymerization, while reductive irradiation in N_2_ yields hydrated electrons to drive anionic polymerization. By controlling the oxidative or reductive irradiation conditions, it is also possible to achieve precise regulation of the PEDOT nanostructure morphology (e.g., spherical or fibrous) and surface properties (hydrophilic or amphiphilic) [40,41]. As an oxidant-free, green approach under mild conditions, radiation polymerization minimizes chemical residues. However, it requires specialized high-energy equipment, incurs high costs, suffers from low batch output, and remains confined to laboratory-scale synthesis.

### 3.5. Molecular Engineering Design

Molecular engineering tailors PEDOT at the monomer or copolymer level to produce functionalized derivatives. EDOT can be modified with functional groups (e.g., alanine, salicylic acid) to impart biocompatibility, chirality, or fluorescence, thereby introducing thermosensitive and other responsive properties into the conductive PEDOT [42]. This strategy enables precise structural and functional customization for advanced applications. However, it involves multi-step synthesis, difficult purification, high costs, sensitive reaction conditions, and low yields, limiting its translation beyond fundamental research.

### 3.6. Standardized Reporting Framework

Poor reproducibility plagues PEDOT research due to undisclosed key parameters. A standardized framework is proposed to unify reporting: (1) PEDOT: PSS mass ratio, governing network structure and mechanics; (2) PSS molecular weight, affecting dispersion and phase separation; and (3) secondary dopant concentration, critical for conductivity enhancement. Auxiliary parameters include solid content, deposition/post-treatment conditions, and humidity. Adherence will enable cross-study comparison and accelerate industrial translation (Table 2).

## 4. Composite Enhancement Mechanism

### 4.1. Mechanism of Composite Synergy

To further improve the performance of PEDOT and maximize its advantageous properties, we did not limit ourselves to using PEDOT as a standalone material; instead, we sought to explore the mechanism of composite synergy to address the shortcomings of the single material. Based on this objective, we conducted specific research. The synergistic mechanism of PEDOT composites involves combining PEDOT with other components—such as inorganic nanomaterials, polymer matrices, and biomolecules—to leverage structural complementarity, performance coupling, and interfacial interactions among these components. This approach overcomes the performance limitations of pure PEDOT (such as poor solubility, insufficient mechanical strength, and limited functionality), achieving a performance enhancement where the whole is greater than the sum of the parts.

#### 4.1.1. Structural Synergy

In the structural synergistic mechanism of PEDOT-based composite materials, the core lies in precisely regulating the aggregation state structure, phase morphology and spatial arrangement of materials through compounding and assembly, so as to simultaneously optimize electrical conductivity and mechanical properties. In the PEDOT: PSS system, insulating PSS chains usually wrap conductive PEDOT segments in a core–shell structure, which hinders carrier transport and limits intrinsic electrical conductivity. The introduction of polar solvents, acidic additives or flexible polymers can effectively regulate the dispersion state and aggregation behavior of PEDOT chains, promote the ordered arrangement of chain segments and the transformation of conjugated structures, and thus greatly enhance electrical conductivity. Carbon nanomaterials such as reduced graphene oxide (rGO), carbon nanotubes (CNTs) and MXene possess the advantages of high conductivity, large specific surface area and high aspect ratio. Their combination with PEDOT can construct a three-dimensional continuous conductive network, greatly shorten the carrier transmission path, and reduce the hopping barrier, achieving an order-of-magnitude improvement in electrical conductivity [47,48].

Taking the modification of silicon-based anodes for lithium-ion batteries as an example, silicon-based particles are co-coated with carbon materials and MXene and then compounded with PEDOT to form a multi-level core–shell structure. This structure not only establishes efficient conductive pathways throughout the electrode but also provides buffer space and mechanical support via the carbon-MXene interlayer to restrain the severe volume expansion of silicon particles during charging and discharging. The outer PEDOT segments achieve ordered arrangement under the regulation of polar environment and interchain interactions, eliminating the obstruction of insulating components to carrier transport. Meanwhile, hydrogen bonding and electrostatic interactions stabilize the electrode structure and maintain the integrity of the conductive network under deformation, which simultaneously solves the key problems of insufficient conductivity and easy structural pulverization of silicon anodes [44,46].

**Figure 4:**
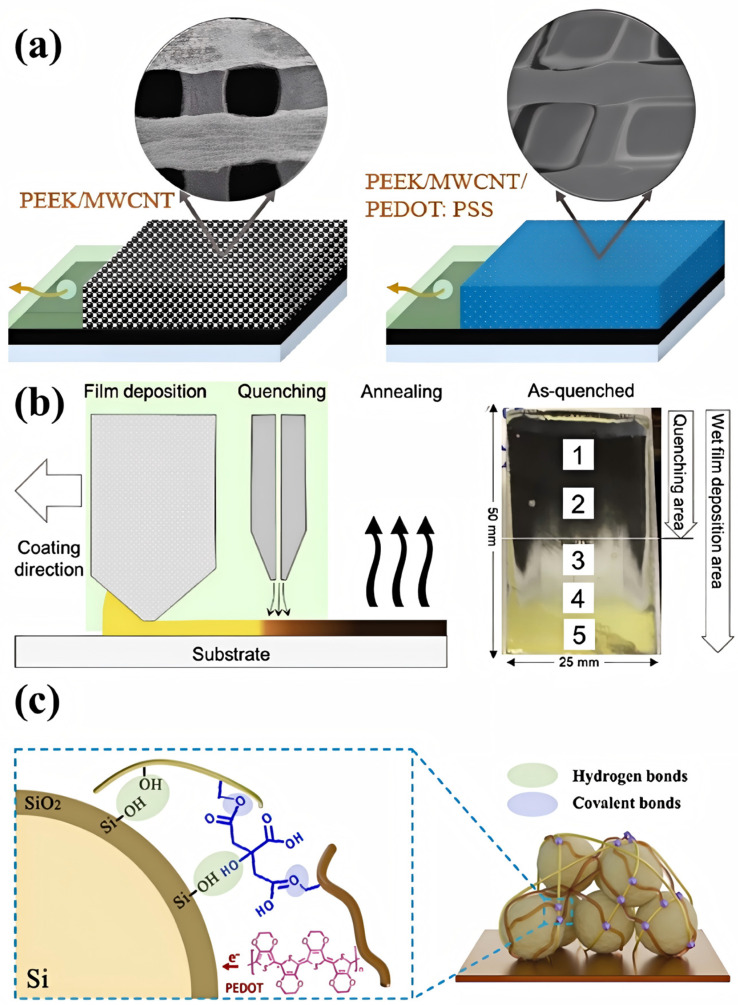
(**a**) These figures demonstrate the full workflow of preparing porous materials through the ice templating (directional freezing) method. (**a**,**b**): PEDOT:PSS (Clevios™ PH1000, Heraeus Holding GmbH, Hanau, Hesse, Germany) scaffolds fabricated using ice-molding technology, exhibiting various shapes such as disks and rectangles; (**b**) Schematic illustration of the preparation process and electrochemical cycling performance of the Si@C-MXene@PEDOT composite anode for lithium-ion batteries. Schematic illustration of the synthesis of the Si-2@C-MXene@PEDOT composite [50]. The as-quenched sample presents five vertical gradient regions. Region 1 at the top is dense solvent-evaporated solid wet film free of precipitates and quenching influence; Region 2 near the quenching boundary retains intact solid film without phase transition; Region 3 at the dividing line serves as the critical transition layer, where quenching triggers phase separation and white flocculent precipitates; Region 4 is pale-yellow liquid hosting dispersed solid precipitates; Region 5 at the bottom refers to pristine pale-yellow stock solution with negligible solute precipitation, forming a top-to-bottom transition from dense solid film to neat solvent. Reproduced with permission, Copyright 2024 Solar RRL. (**c**) Schematic illustration of the complete preparation process for the PEDOT/Ti_3_C_2_T_x_/Co_3_S_2_ ternary composite electrode material. Shows the 3C2Tx etching process for Ti, as well as the loading processes for cobalt sulfide and PEDOT [38]. Reproduced with permission, Copyright 2024 Energy & Environmental Materials.

In addition, blending PEDOT with flexible polymers such as polyvinyl alcohol (PVA) and polyurethane can form uniform phase-separated structures through hydrogen bonding and electrostatic interactions, improving film-forming ability, ductility and environmental stability while boosting electrical conductivity. The hydrogen bond network formed between PVA and PEDOT: PSS further endows composite films with high strength and superior flexibility, laying a solid material foundation for stretchable electronic devices (Figure 4) [46,49].

#### 4.1.2. Performance Coupling and Synergy

Performance coupling synergy emphasizes the functional complementarity of different components, enabling composite materials to integrate multiple properties such as electrical, mechanical, thermal, and electrochemical performances, thereby breaking through the performance limits of single-component materials. The dual hydrogen-bond network formed by PEDOT: PSS with PVA and citric acid endows the material with high tensile strength, self-healing properties, and excellent thermoelectric performance while maintaining high conductivity and has been successfully applied in wearable strain sensors, temperature sensors, and human–machine interfaces [34]. Such electrical–mechanical performance coupling is the key to the practical application of flexible electronic devices. The composite of PEDOT: PSS with rGO/CNT aerogels not only improves electrical conductivity but also further optimizes thermoelectric conversion efficiency through interfacial interactions, providing a feasible solution for organic thermoelectric devices. Taking the PEDOT/Ti_3_C_2_T_x_/Co_3_S_2_ ternary composite electrode material as an example, this system achieves functional coupling of multiple components through layer-by-layer integration: MXene (Ti_3_C_2_T_x_) provides a highly conductive two-dimensional skeleton, Co_3_S_2_ contributes high pseudocapacitive properties, and the outer PEDOT constructs a continuous conductive coating layer. The synergistic effect of the three components endows the material with high conductivity, high specific surface area, and high redox activity simultaneously, realizing the synchronous improvement of specific capacity, rate performance, and cycling stability, which perfectly fits the performance coupling mechanism of ternary composite energy storage systems [36].

#### 4.1.3. Interface Integration

Interfacial synergy is a key factor determining the final performance of composite materials, mainly manifested in the physical and chemical interactions at the interfaces of different components, including π–π interactions, hydrogen bonds, chemical bonds, and charge transfer. Carbon-based nanofillers can form strong interactions with PEDOT through π–π stacking, which not only ensures stable dispersion but also guides the oriented crystallization of PEDOT chains, optimizes the phase separation scale, and provides fast carrier transport channels. In the preparation process of the PEDOT/Ti_3_C_2_T_x_/Co_3_S_2_ ternary system, the interfacial synergy effect runs through the entire process: first, Ti_3_AlC_2_ is etched with HCl and LiF to obtain Ti_3_C_2_T_x_, providing a two-dimensional substrate with abundant surface functional groups for subsequent composite; then, Co_3_S_2_ is anchored on the Ti_3_C_2_T_x_ sheets via a solid-phase heating method to form a stable heterogeneous interface; finally, PEDOT is uniformly grown and coated on Ti_3_C_2_T_x_/Co_3_S_2_ through the (2,5-dibromo-3,4-ethylenedioxythiophene) DBEDOT process, forming strong interactions with MXene via π–π stacking and hydrogen bonds, while establishing a close contact interface with Co_3_S_2_. Such multiple interfaces formed among PEDOT, MXene, and metal sulfides in the ternary electrode material promote rapid electron transfer and sufficient ion contact, significantly improving electrochemical activity and structural stability [36]. The hydrogen bonding between PEDOT: PSS and polymers such as PVA is a classic example of polymer–polymer interfacial synergy. This interaction not only enhances mechanical properties but also regulates chain motion and aggregation modes, keeping the conductive network stable under deformation and providing a key strategy for the design of highly stretchable conductive films (Figure 4) [35].

#### 4.1.4. Doping Synergy

Doping synergistically modulates the electronic structure and aggregation state of PEDOT chains through small molecules, ions, or solvents, thereby increasing carrier concentration and mobility. It is the most direct and commonly used method for enhancing electrical conductivity. The addition of acidic dopants to PEDOT: PSS promotes the entanglement of PSS chains, allowing PEDOT chains to pack more tightly and form continuous, highly efficient conductive pathways, thereby increasing electrical conductivity by several orders of magnitude [32] (Figure 4). The incorporation of salts and polar solvents can further modulate chain conformation and aggregation behavior, enabling a transition from nonlinear to linear conductive response and expanding their applications in sensing and flexible circuits [35]. By incorporating PEDOT into a mesoporous nanocarbon matrix to form a composite electrode and leveraging nanoscale confinement and interfacial interactions, capacitive characteristics and rate performance can be significantly enhanced, providing a new design approach for high-performance aqueous symmetric supercapacitors [37].

#### 4.1.5. Biological Synergy

Bio-synergistic Focus highlights the compatibility and functional integration between PEDOT-based materials and biological systems, emphasizing their unique advantages in bioelectronics, tissue engineering, and implantable devices. PEDOT inherently possesses excellent biocompatibility and electrochemical activity; when combined with biocompatible materials such as PVA, it becomes even more suitable for applications in biosensors, neural interfaces, and flexible electrodes [32]. Its high electrical conductivity, low electrical resistance, ease of processing, and high flexibility make it an ideal candidate material for wearable and implantable electronic devices, including neural probes, artificial muscles, and in vivo physiological signal monitoring systems [32,33]. By modulating cell behavior and promoting nerve growth and tissue repair through electroactivity, PEDOT-based composites can interact synergistically with bioactive molecules and scaffold materials to integrate electrical signal transmission, controlled drug release, and tissue regeneration, demonstrating broad prospects in the fields of tissue engineering and regenerative medicine [32] (Figure 4).

## 5. Research on the Interaction of PEDOT with Other Substances

### 5.1. Combination of PEDOT and Conductive Polymers

As mentioned above, PEDOT exhibits excellent electrochemical activity and environmental stability; however, when used as a single component, it still faces inherent limitations in practical applications, such as limited charge storage capacity, significant mechanical brittleness, and processing constraints. Composites formed by blending PEDOT with other typical conductive polymers, such as polyaniline (PANI) and polypyrrole (PPy), aim to simultaneously optimize charge transport efficiency, electrochemical activity, and mechanical stability through the synergistic construction of interpenetrating networks or ordered nanostructures by multiple components. The core scientific question underlying this compositing strategy is as follows: how do molecular-level interactions between different conductive polymer segments reorganize the aggregate structure of PEDOT, thereby synergistically enhancing the material’s electrochemical and mechanical properties [51,52,53]?

From the perspective of the binding mechanism, the composite formation of PEDOT and a second conductive polymer follows two typical patterns. The first is template-induced growth: using PEDOT as a substrate, its counterions (such as PSS^−^) or surface functional groups serve as nucleation sites to induce the oriented polymerization of PANI, PPy, and other polymers on its surface, forming vertical arrays or core–shell structures. During this process, the conjugated backbone of PEDOT strongly couples with the π-electron system of the second polymer, stabilizing the heterojunction through π–π stacking and electrostatic interactions. The second approach involves the construction of interpenetrating networks: through in situ polymerization or solution blending, PEDOT segments and second-polymer segments intertwine and entangle to form a three-dimensional continuous conductive network. Both modes aim to optimize charge transport pathways—the former enhances kinetic performance by shortening the ion diffusion distance, while the latter reduces bulk resistance by filling defects in the conductive pathways.

The introduction of a second conductive polymer significantly alters the intrinsic structure of PEDOT [54,55]. At the molecular chain conformation level, Raman spectroscopy revealed that the C=C stretching peak of PEDOT after compounding shifted from 1436 cm^−1^ (benzene structure) to 1428–1410 cm^−1^ (quinone structure), confirming an increase in the conjugation of the main chain and enhanced carrier delocalization. At the aggregate level, X-ray diffraction (XRD) indicates that the half-width at half-maximum (HWHM) of the composite’s diffraction peaks narrows (e.g., from 0.65° to 0.48°), with a significant increase in crystallinity. This is attributed to the second polymer segment inducing an ordered rearrangement of the PEDOT molecular chains. At the electronic structure level, the characteristic PEDOT peaks (e.g., S 2p) in X-ray photoelectron spectroscopy (XPS) shifted by 0.3–0.5 eV, demonstrating charge transfer between the two phases and optimized interface energy level matching.

**Figure 5:**
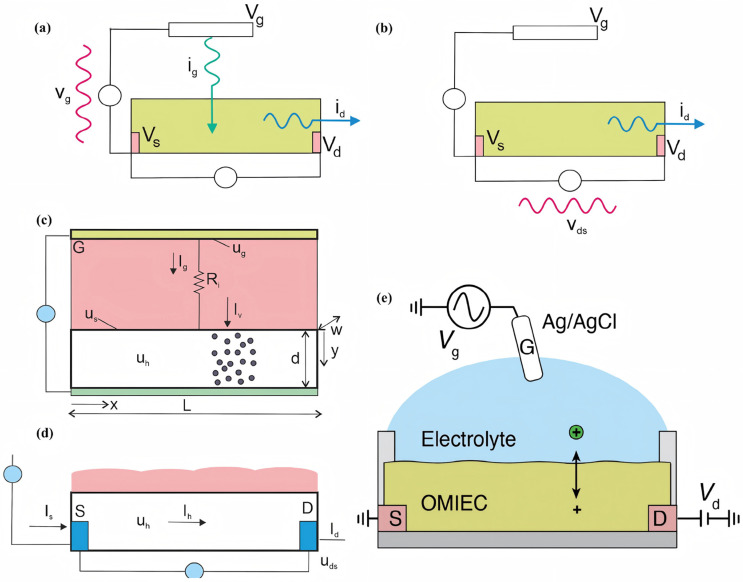
This figure illustrates the preparation process of PANI and PEDOT/PANI-based electrodes, the structure of a flexible supercapacitor, and its electrochemical performance including cyclic voltammetry and galvanostatic charge–discharge curves. (**a**) Schematic illustration of the preparation process for the PANI:HSO_4_^−^ and PEDOT/PANI:(HSO_4_^−^)_x_:(CF_3_SO_3_^−^)_1−x_ electrodes [58]. (**b**) Schematic diagram of a flexible supercapacitor based on the PEDOT/PANI:(HSO_4_^−^)_x_:(CF_3_SO_3_^−^)_1−x_ electrode [58]. (**c**) Cyclic voltammetry curves of the flexible supercapacitor at different scan rates ranging from 10 to 100 mV·s^−1^ [58]. (**d**,**e**) Constant-current charge–discharge curves of the flexible supercapacitor at current densities ranging from 2 to 30 A·g^−1^ [58]. Reproduced with permission, Copyright 2024 Colloids and Surfaces A: Physicochemical and Engineering Aspects.

The significant improvement in electrochemical performance is a direct manifestation of this synergistic effect. The assembled flexible supercapacitor achieved a specific capacitance of 361.6 F·g^−1^; after 10,000 cycles, the capacitance retention rate was 71%, and the energy density was 28.5 mWh·g^−1^ at a power density of 789.5 mW·g^−1^ (Figure 5). Figure 5 systematically demonstrates the fabrication process of PEDOT/PANI composite electrodes, the structure of the flexible supercapacitor, and its electrochemical performance. Part (a) shows the preparation of PANI: HSO_4_^−^ single-layer electrodes and PEDOT/PANI:(HSO_4_^−^)_x_(CF_3_SO_3_^−^)_1−x_ composite electrodes via a two-step polymerization process, achieving morphological control from disordered granular structures to oriented nanorod arrays. Part (b) presents the structure of the flexible supercapacitor using PVA-H_2_SO_4_ as the gel electrolyte, clearly illustrating the electrode layers and ion transport pathways. Parts (c) and (d) display the cyclic voltammetry curves at different scan rates and galvanostatic charge–discharge curves at various current densities, respectively, verifying the excellent rate capability and capacitive reversibility of the composite electrodes. This provides intuitive support for the structural design and performance optimization of flexible energy storage devices. Notably, the PEDOT/PANI nanorod array structure enabled the electrode to exhibit a resistance change of less than 5% after 1000 cycles at a bending radius of 5 mm, demonstrating its excellent mechanical flexibility. The improvement in mechanical properties stems from the physical cross-linking of PEDOT segments by the interpenetrating network formed by the second polymer [39,56,57]. In the PEDOT/PPy system, the three-dimensional network formed by in situ polymerization effectively suppresses excessive entanglement and stress concentration of the PEDOT chains, resulting in a more uniform distribution of the conductive components (EDS elemental mapping shows a correlation coefficient of 0.92 between sulfur and nitrogen distributions). At the same time, external forces are dissipated through interchain π–π interactions, enabling the composite fabric to maintain the integrity of its conductive network even after repeated bending. The composites of polyaniline (PANI) and polypyrrole (PPy) exhibit remarkable advantages, including a significantly enhanced specific capacitance (1042.7 F·g^−1^) and a more continuous conductive network. However, they suffer from inherent drawbacks: poor interfacial compatibility between the two polymers leads to easy delamination after long-term cycling and weak mechanical stability. Additionally, there exists a substantial variation in the composite ratios across different studies, resulting in no consensus on the optimal ratio. Furthermore, the interfacial interactions are mostly described phenomenologically, lacking sufficient support from in situ characterization evidence.

### 5.2. Combination of PEDOT with Non-Conductive Polymers

The primary objective of this research is to elucidate how non-conductive polymers (such as polyvinyl alcohol (PVA), polyethylene oxide (PEO), and polymethyl methacrylate (PMMA)) modulate the microstructure of PEDOT: PSS composites through molecular-level interactions and, based on this, to clarify the mechanisms by which such modulation influences the formation of conductive networks, carrier transport pathways, and macroscopic mechanical integrity.

**Figure 6:**
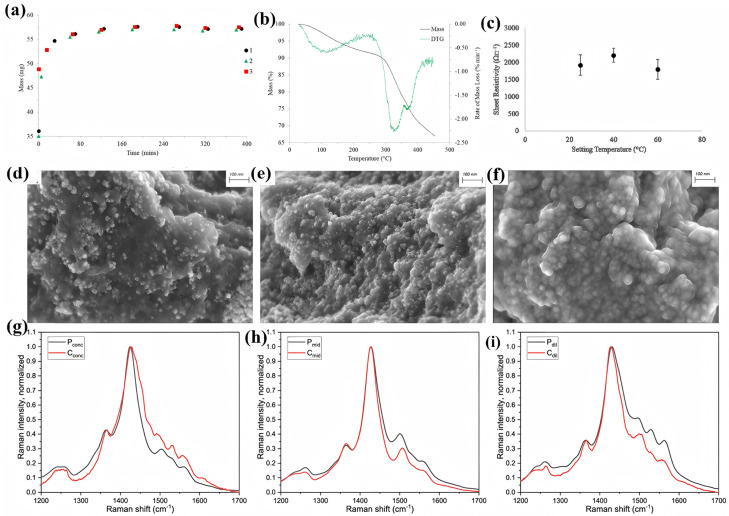
Demonstrates the synergistic mechanism of the PEDOT–metal composite (**a**) Raman spectra [49]. (**b**) S 2p XPS spectra of PEDOT:PSS fibers prepared from different solidification baths [49]. (**c**) Conductivity histogram [49]; reproduced with permission, Copyright 2021 *Composites Communications*. (**d**–**f**) Scanning electron microscope (SEM) images of PEDOT composites; Raman spectra of pure PEDOT samples and PEDOT:CoFe_2_O_4_ composites, with an excitation wavelength of λ_exc_ = 633 nm [25]. (**g**) Synthesis in concentrated solution [25]. (**h**) Synthesis in medium-concentration solution [25]. (**i**) Synthesis in dilute solution. All spectra are normalized to their respective peaks [25]. Reproduced with permission, Copyright 2025 *Polymer*.

The compatibility of PEDOT:PSS with non-conductive polymers primarily depends on multiple secondary interactions. Among these, the polystyrene sulfonic acid (PSS^−^) component plays a key role as a “compatibilizer” [40,41]. The sulfonic acid groups on the PSS^−^ chain can form strong hydrogen bonds or dipole–dipole interactions with non-conductive polymers (such as hydroxyl groups in PVA or ether oxygen groups in PEO). At the same time, the π-electron cloud of the PEDOT main chain may also interact with the polar groups of non-conductive polymers through weak van der Waals forces or dipole-π interactions. These synergistic interactions effectively bridge the originally incompatible conductive and insulating phases, promoting the uniform dispersion of PEDOT-enriched nanodomains within the non-conductive polymer matrix and suppressing excessive aggregation of PEDOT:PSS particles, thereby forming a more continuous and efficient charge transport network [42,43]. These molecular-level interactions have caused quantifiable changes in the structure of PEDOT itself. X-ray diffraction (XRD) analysis indicates that the introduction of non-conductive polymers generally reduces the crystallinity of PEDOT by approximately 15–25%, as evidenced by a significant increase in the half-width of the (100) plane diffraction peak in the XRD pattern [44] (Figure 6c,d). Glancing-incidence wide-angle X-ray scattering (GIWAXS) data further confirm that the π–π stacking distance between PEDOT main chains has increased slightly from approximately 3.6 Å in pure PEDOT:PSS films to 3.7–3.8 Å [45]. It is worth noting that, despite the change in crystal structure, the electronic structure of the PEDOT backbone remains stable. In X-ray photoelectron spectroscopy (XPS), the binding energy peak of the S2p_3_/_2_ orbital remains at approximately 168.2 eV without any significant shift, indicating that the composite process did not induce chemical doping or de-doping of PEDOT, and its high conductivity is thus preserved.

In terms of electrochemical performance, the composite system exhibits typical trade-offs and optimization characteristics. As mentioned earlier, on the one hand, the specific capacitance of the composite material typically decreases due to the diluting effect of the non-conductive polymer. For example, in a three-electrode system, measured using 0.1 M PBS as the electrolyte at a scan rate of 10 mV s^−1^, the specific capacitance of the PEDOT:PSS/PEO (70:30 *w*/*w*) composite film was 89 ± 6 F g^−1^, which is lower than the 115 ± 8 F g^−1^ of the pure PEDOT:PSS film [45]. However, on the other hand, ion transport kinetics have been significantly improved. At a high scan rate of 100 mV s^−1^, the capacitance retention of this composite material reached as high as 76%, whereas that of pure PEDOT:PSS was only 52% [45]. Electrochemical impedance spectroscopy (EIS) analysis provides direct evidence of this: the charge transfer resistance (Rct) of the composite decreased from 42 Ω for pure PEDOT: PSS to 27 Ω, indicating a significant reduction in the diffusion impedance of ions at the electrode/electrolyte interface. This is attributed to the more open and hydrophilic porous network structure formed by the introduction of the non-conductive polymer, which facilitates the rapid penetration and migration of electrolyte ions.

In terms of mechanical properties, the combination of PEDOT with non-conductive polymers has led to a revolutionary improvement. As a flexible matrix, the non-conductive polymer effectively absorbs and dissipates external stress, enhancing the toughness and ductility of the composite material. Taking the PEDOT:PSS/PVA (60:40 *w*/*w*) blend film as an example, its tensile strength significantly increased from 12.3 ± 0.9 MPa in pure PEDOT:PSS film to 38.7 ± 2.1 MPa [44]. Most notably, its elongation at break surged from a brittle 3.1 ± 0.4% to 142 ± 11%, marking a transition from brittle to highly elastic behavior [44]. At the same time, the Young’s modulus decreased from 1.8 ± 0.2 GPa to 0.36 ± 0.03 GPa, indicating that the material became less rigid and more flexible [44]. This combination of mechanical properties, a balance of strength and flexibility, which involves the simultaneous enhancement of both strength and ductility, is unattainable with PEDOT: PSS alone and provides a solid mechanical foundation for the long-term stable operation of flexible electronic devices. The PVA/PEO composites exhibit remarkable advantages, including a substantial enhancement in ductility (from 3.1% to 142%) and a significant improvement in flexibility. However, inherent drawbacks exist: the insulating nature of non-conductive polymers dilutes the conductive network, which in turn leads to a noticeable decrease in electrical conductivity and a reduction in electrochemical activity. Current research efforts predominantly focus on optimizing mechanical properties while neglecting the inherent trade-off between conductivity and mechanical performance. This oversight renders it challenging to simultaneously achieve the dual requirements of high electrical conductivity and excellent flexibility, which are crucial for advanced functional applications.

### 5.3. Combination of PEDOT with Metal Oxides

In addition to combining with conductive or non-conductive polymers, the integration of PEDOT with metallic materials is currently a major area of research. This approach reveals how metallic materials regulate the microstructure and charge transport behavior of PEDOT and its composite systems through multiple mechanisms, including interfacial charge transfer, coordination complexation, and template effects, thereby influencing the formation of conductive networks, electrochemical activity, and structural stability [25,46,59].

**Figure 7:**
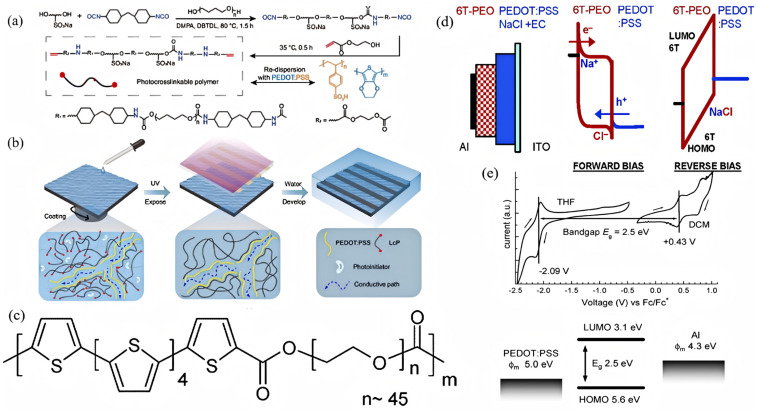
Two types of PEDOT:PSS devices are presented: one by Matrone et al. [60] simulating neurotransmitter-mediated synaptic plasticity, the other by Verbakel et al. [61] demonstrating the synergy between rectification and switching behavior. (**a**) Schematic illustration of the ENODe’s gate electrode, as well as the combination of the fluidic module and the channel outlet, corresponding to the presynaptic and postsynaptic terminals of a biological synapse, respectively [62]. (**b**) Channel conductance modulation GD (ii) and integral optical density change ∫OD (iii) were measured during gate-period voltage pulses (i) (three square pulses with a duration of 3 s and a 10 s delay) that triggered neurotransmitter oxidation (static PBS neurotransmitter solution in the fluidic module), DA (left, red) and 5-HT (right, blue) undergo changes, followed by a return to baseline. Input is modulated by three columns of pulses (0.1 to 0.8 Vg, ∆Vg 0.1 V, Vd −0.1 V) under a PBS flow rate of 1 mL/min [62]. Reproduced with permission, Copyright 2023 *Advanced Materials Technologies*. (**c**) Chemical structure of 6T-PEO block copolymers [61]. (**d**) Schematic layout of the ITO/PEDOT: PSS (NaCl, EC)/6T-PEO/Al device (left) and band diagrams of the diode under forward bias (middle) and reverse bias (right). Indicates the injection of negative (**e**) and positive.

From the perspective of binding mechanisms, the interaction between PEDOT and metallic materials exhibits diverse characteristics. Taking cobalt ferrite (CoFe_2_O_4_) magnetic nanoparticles as an example, their surfaces can induce the selective adsorption of PEDOT monomers. During the chemical oxidation–polymerization process, interfacial effects alter the conjugated length, oxidation state, and resonance structure of PEDOT, thereby influencing the formation and arrangement of polymer chains (Figure 7d,e) [25]. In metal salt systems (such as LiCl and CaCl_2_), metal ions (Li^+^ and Ca^2+^) can form electrostatic complexes with the sulfonic acid groups on the PSS^−^ chains (Figure 7b). Through cross-linking effects, this induces the reorganization and stabilization of PEDOT:PSS segments, enabling the continuous formation of fibers in the coagulation bath [59]. In nickel fiber network (NiSFs) composite systems, the three-dimensional conductive network provides fast electron transport pathways, but the interfacial charge transfer process is governed by the potential barrier at the metal–polymer interface [46]. These synergistic effects effectively bridge the conductive polymer and the metal material, promoting the ordered distribution of PEDOT-enriched regions on the surface of the metal substrate or within the interstitial spaces of the network, thereby forming a multiscale charge transport network. The introduction of the metal material induces quantifiable changes in the intrinsic structure of PEDOT. Raman spectroscopy analysis indicates that the introduction of CoFe_2_O_4_ nanoparticles induces a transition of the PEDOT main chain from a benzene-like conformation to a quinone-like conformation, resulting in a red shift and narrowing of the Cₐ=Cᵦ symmetric stretching vibration peak, as well as a significant increase in conjugated length [25]. X-ray diffraction (XRD) further confirmed that metal nanoparticles act as nucleation sites to induce ordered alignment of PEDOT chains; at low reactant concentrations, the electrical conductivity of the PEDOT regions adjacent to the nanoparticles increased by more than two orders of magnitude (up to 600-fold) compared to pure PEDOT [47]. Notably, X-ray photoelectron spectroscopy (XPS) revealed no significant shift in the binding energy peak of the PEDOT S2p orbital, indicating that the composite formation did not alter its intrinsic doped state and that the chemical structure remained stable. For the metal salt-modified system, small-angle X-ray scattering (SAXS) revealed the formation of an anisotropic structure within the PEDOT:PSS fibers, with PEDOT-enriched regions preferentially oriented along the fiber axis, providing a more efficient pathway for carrier transport [59].

The optimization of electrochemical performance exhibits system dependence. In the Ag nanoparticle-doped PEDOT:PSS system, the specific capacitance of the composite exhibits a sharp increase with increasing Ag content; the AgNPs-PEDOT:PSS 15% sample demonstrates the best capacitive performance, which is attributed to the effective charge storage at the metal–polymer interface layer [48]. However, the charge transfer resistance (Rct) of the nickel fiber network/PEDOT:PSS composite electrode increased from 131.5 Ω for pure PEDOT:PSS to 170.9 Ω, indicating that the introduction of nickel fibers slowed down interfacial charge transfer to some extent; this is attributed to the hindrance of charge injection caused by the metal/polymer interfacial barrier [46]. Nevertheless, while the bandgap (Eg) of this composite material has decreased slightly from 1.34 eV to 1.31 eV, its optical properties remain largely unchanged, and it still holds potential for application in optoelectronic devices [46]. The increase in electrical conductivity of the cobalt ferrite composite system is closely related to the concentration of reactants; under optimized conditions, it can be more than two orders of magnitude higher than that of pure PEDOT [25].

In terms of mechanical properties, the incorporation of metallic materials results in significant strengthening and toughening effects. The electrostatic coordination of Li^+^ effectively bridges PSS segments, significantly increasing the elongation at break of PEDOT:PSS wet-spun fibers from less than 5% to over 50% (one of the highest values reported to date) (Figure 7a), while simultaneously increasing the electrical conductivity from 215 S/cm to 807 S/cm (Figure 7c), thereby achieving simultaneous optimization of mechanical flexibility and electrical performance [59]. This synergistic enhancement effect, characterized by a balance of stiffness and flexibility, stems from the rearrangement of PEDOT chains and the formation of an anisotropic structure induced by metal ion cross-linking. In the Fe^3+^-cross-linked sodium alginate/PEDOT hydrogel system, the metal coordination bonds not only provide high elasticity to dissipate energy but also endow the composite with a conductivity of 0.32 S/cm, an elastic modulus of 550 MPa, a tensile strength of 0.81 MPa, and a tensile strain at break of 473%, demonstrating comprehensive mechanical properties that are difficult to achieve with PEDOT alone. The composites of metals (e.g., gold, silver, copper) and PEDOT can significantly enhance electrical conductivity, construct a continuous conductive network, and exhibit remarkable conductive enhancement effects. However, metal nanoparticles are highly prone to agglomeration, which impairs the structural uniformity of PEDOT and thus leads to performance fluctuations. Meanwhile, metal materials suffer from poor biocompatibility, high susceptibility to inducing immune responses, and insufficient long-term stability, which severely limit their applications in the field of implantable bioelectronics and restrict their use to non-biological scenarios only.

### 5.4. Combination of PEDOT with Inorganic Nanoparticles and Biomaterials

Finally, PEDOT is combined with inorganic nanoparticles and biomaterials. This composite is typically used in applications as a bioelectrode or a vehicle for drug delivery; this topic will be discussed in detail in subsequent sections. The key to this combination lies in elucidating how the plasmonic resonance and quantum confinement effects of the inorganic nanoparticles, as well as the functional groups of the biomaterials, modulate the aggregate structure, charge transport behavior, and biocompatibility of PEDOT.

Inorganic nanoparticles primarily rely on interfacial charge transfer and template-assisted assembly. The surface plasmon resonance of gold and silver nanoparticles enhances the localized electromagnetic field, promoting the generation of PEDOT charge carriers; tellurium quantum dots, on the other hand, are tightly encapsulated by PEDOT (particle size < 5 nm) due to the self-inhibition effect of the Fe(III) oxidant, achieving a precise loading of 2.1–5.8 wt% [63]. The assembly of biomaterial composites relies on secondary interactions: after copolymerization of hyaluronic acid (HA) with PEDOT, its carboxyl groups can undergo electrostatic layer-by-layer assembly with poly-L-lysine (PLL); hydroxyl groups on the surface of chitin nanocrystals (ChNCs) guide the in situ polymerization of PEDOT via hydrogen bonding, forming core–shell nanostructures [64,65]. The introduction of inorganic nanoparticles and biomaterials induces quantifiable changes in the PEDOT structure. Raman spectroscopy reveals that nanoparticles induce a transition of the PEDOT main chain from a benzene-like to a quinone-like structure, with a significant increase in conjugated length; the incorporation of tellurium quantum dots results in a substantial reduction in the lattice thermal conductivity of the composite film while maintaining high electrical conductivity. XPS analysis confirms that secondary doping with vitamin C effectively removes excess PSS, resulting in a significant increase in the peak area ratio of PEDOT to PSS; the uniform distribution of nitrogen in the PEDOT: HA/PLL system confirms the presence of electrostatic interactions.

The optimization of electrochemical performance exhibits system dependence. Au/Ag bimetallic-doped PEDOT: PSS demonstrates excellent broad-spectrum response sensitivity in self-powered photodetectors. PEDOT-Au nanoparticle-modified electrodes demonstrate high-sensitivity detection capabilities for dopamine. Secondary doping with vitamin C significantly enhances the specific capacity and conductivity of PEDOT: PSS; the assembled devices exhibit high-capacity retention after long-term cycling, and their hemolysis rate is well below the safety threshold. PEDOT/ChNCs composites achieve good electrochemical energy storage performance while maintaining sustainability.

Improved biocompatibility and mechanical properties are the standout advantages of this class of composite systems. PEDOT: HA nanoparticle-reinforced PAA/HA/lignin scaffolds significantly enhance cell viability. The energy storage modulus of alginate-laminins-PEDOT: PSS hydrogels closely match that of neural tissue, and they exhibit excellent optical transparency. PEDOT: HA/PLL multilayer films significantly enhance the compressive strength of the scaffold and reduce its swelling rate. After implantation into mice, vitamin C-doped supercapacitors showed no significant adhesion between their surface and surrounding tissues; hematological and histological analyses confirmed their long-term biocompatibility. Key performance evaluation metrics: Composites of inorganic nanomaterials (carbon nanotubes, graphene, MXene) with PEDOT can achieve a balanced integration of electrical conductivity, mechanical properties, and stability, featuring well-rounded comprehensive performance that is compatible with diverse application scenarios. However, inorganic nanoparticles possess a large specific surface area and high surface energy, which render them highly prone to agglomeration and poor dispersibility in the PEDOT matrix, making it difficult to form a uniform composite structure. Additionally, complex surface modification is required, which leads to cumbersome processes and increased costs, thereby hindering large-scale fabrication.

### 5.5. Comparison of PEDOT with Other Substances

Existing PEDOT composite systems exhibit obvious performance trade-off characteristics: composite with conductive polymers can significantly improve energy storage capacity but suffers from insufficient interface stability and limited cycle life; composite with non-conductive polymers can endow the material with excellent flexibility and stretchability, yet inevitably impairs the conductive network and causes a decrease in electrical conductivity; metal/inorganic composites can achieve a balanced performance in conductivity, mechanics, and stability, but are hindered by complex preparation processes, high costs, and great difficulties in large-scale production; and composite with biomaterials possesses excellent biocompatibility, making it applicable to bioelectronic scenarios, but generally has the shortcomings of weak electrical conductivity and poor electrochemical stability. Overall, current research mostly remains at the level of simple component superposition and macro-performance characterization, lacking systematic analysis of microcosmic synergetic mechanisms such as interface interactions, electron transfer, and structural reconstruction. A clear structure–activity relationship has not yet been established, which restricts the rational design and performance breakthrough of high-performance PEDOT composites (Table 3).

## 6. Applications of PEDOT and PEDOT: PSS CPH in Combination with Other Materials

### 6.1. Applications of PEDOT

#### 6.1.1. Applications of PEDOT in High-Performance Sensors and Data Transmission

Addressing the challenges associated with the complex fabrication process and functional synergy of PEDOT-based flexible multimodal sensors, the team led by Wu Pingping further coupled a supramolecular conductive composite membrane electrode with a dome-shaped microstructure to a PVDF-HFP dielectric layer doped with an ionic liquid (EMIM: TFSI) and featuring a pyramid-shaped microstructure. In fitness and sports, continuous and accurate temperature tracking helps to optimize performance and prevent heat-related illnesses [66]. By synergistically regulating the double-layer capacitance mechanism and the microstructural contact interface, the sensor achieved ultra-high sensitivities of 1756 kPa^−1^, 556 kPa^−1^, and 225 kPa^−1^ in the pressure ranges of 0–50 kPa, 50–150 kPa, and 150–400 kPa, achieving ultra-high sensitivities of 1756 kPa^−1^, 556 kPa^−1^, and 225 kPa^−1^ respectively. Furthermore, thanks to the high electrical conductivity and unique layered structure of the supramolecular conductive composite film, the device also exhibits excellent electromagnetic shielding performance exceeding 50 dB. The SCCFs studied by the researchers exhibit ultra-low modulus, high conductivity, and good adhesion. Clearly, compared to other materials with low modulus, high conductivity, and strong adhesion, their SCCF maintains high performance across all three dimensions [67,68,69,70,71,72,73]. In contrast to Wu Pingping’s team, who used a DMSO-mediated co-induction strategy to construct a bilayer film based on PEDOT:PSS and PVDF(HFP) and achieved high-precision recognition of strain–pressure–humidity triple sensing and high-precision recognition via machine learning, Yin Ao and colleagues focused on pushing the performance limits of electrode materials through supramolecular chemistry and microstructural engineering, elevating the sensitivity and detection range of capacitive tactile sensors to new heights while endowing them with multi-directional force detection and electromagnetic shielding capabilities. By addressing functional integration and performance optimization, these two studies provide complementary innovative approaches for the design of flexible multimodal sensing systems. In fact, the widespread application of PEDOT:PSS in fields such as flexible [74,75,76] and stretchable electronics [74,75,76,77], bioelectronics [77] and high-performance sensors [78] is based precisely on its excellent electrical conductivity, mechanical flexibility and processability; these properties make PEDOT-based materials ideal candidates for flexible multimodal sensing systems.

Despite the remarkable progress achieved in the sensitivity and multimodal integration of PEDOT-based sensors, the performance parameters across different studies remain difficult to directly compare due to variations in testing conditions. Furthermore, the simultaneous achievement of ultra-high sensitivity and long-term cyclic stability remains a persistent challenge. Future research should focus on verifying the reliability of these devices under complex real-world environments.

#### 6.1.2. Applications of PEDOT and Its Polymers in Printing and Pattern Formation

**Figure 8:**
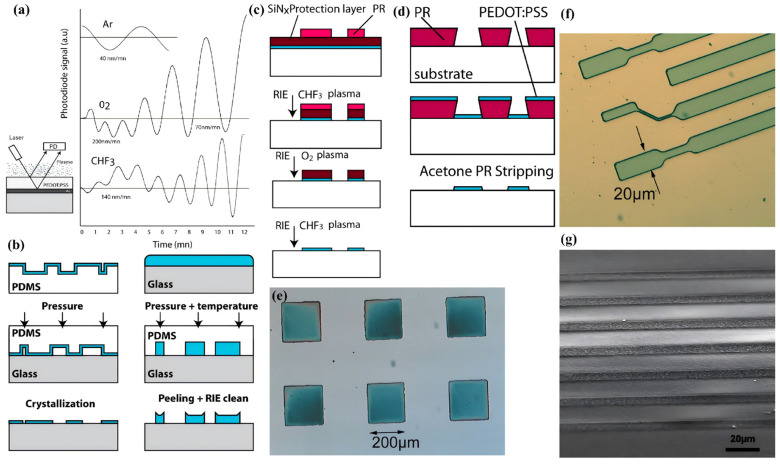
A variety of PEDOT: PSS patterning methods are demonstrated: fluorine-based gas etching, soft lithography and casting techniques, and conventional peeling methods; meanwhile, a new process based on a silicon nitride protective layer overcomes chemical incompatibility and yields micrometer-scale patterns with well-defined edges. (**a**) Analysis of RIE ICP etching rates using interferometric measurements; the period of the photodiode signal corresponds to the etching depth λ/2n [84]. (**b**) Micro-contact printing and casting process on PDMS scaffolds [84]. (**c**) Process flow for protective coating technology [84]. (**d**) The process of de-linking technology [84]. (**e**) Microscopic photograph of the PEDOT-PSS glass pattern obtained using an improved technique [84]. (**f**) Micrograph of a patterned PEDOT:PSS structure fabricated using photolithography with a silicon nitride protective layer [84]. (**g**) SEM image of a 20-micrometer-wide PEDOT-PSS line formed via photolithography and a silicon nitride protective layer [84]. Reproduced with permission, Copyright 2013 *Microsystem Technologies*.

Current research on PEDOT and its polymers in the field of printing and patterning primarily focuses on the challenge posed by the sensitivity of PEDOT:PSS films to common chemicals and moisture, which makes it difficult to use standard photolithography processes for micrometer-scale patterning [79,80,81,82,83]. Charlot and colleagues conducted a systematic review of various patterning strategies [84]. The study also proposed a novel photolithography process based on a silicon nitride protective layer. The research first evaluated the etching behavior of PEDOT:PSS under different plasma etching gases and found that the sputtering etching rate of argon was relatively low (20–40 nm/min) [85]. The initial etching rate of oxygen plasma can reach 400 nm/min, but it decreases to 70 nm/min over time [86]. Meanwhile, the etching rate of the CHF_3_-based fluorine gas remains stable at 140 nm/min [84]. This indicates that fluorinated gases are better suited for precise control of etching depth (Figure 8a). In terms of soft lithography, the study demonstrates a method for fabricating stretchable electrodes by filling polydimethylsiloxane (PDMS) microchannels with a PEDOT: PSS solution [87,88,89]. Conductive connections can be formed in 200 μm wide grooves through multiple filling and thermal crystallization cycles; simultaneously, a micro-contact printing method based on PDMS stamps has been developed [79,80,90]. Using casting technology (Figure 8b), a PEDOT:PSS pattern with a depth of 80 μm can be obtained, but additional oxygen plasma etching is required to remove the residual thin layer [84]. In the field of traditional lithography, the study compared the peel-off method [91,92,93] (Figure 8d) and the results of the protective layer method: In the stripping method, PEDOT:PSS is spin-coated after patterning with positive photoresist, and the pattern is obtained by dissolving the photoresist with acetone; however, the resulting lines have rough edges and a U-shaped cross-section (Figure 8e) [84,94]. To this end, the study proposes a new process that uses PECVD to deposit a 100 nm thick silicon nitride layer as a protective layer (Figure 8c) [95,96]. This protective layer prevents direct contact between the photoresist developer and PEDOT: PSS. Through precise control of plasma etching using CHF_3_ and O_2_, we successfully obtained 20 μm wide PEDOT: PSS microclines with sharp edges and regular shapes (Figure 8f,g). Since the SiN protective layer can be deposited at low temperatures (100–200 °C) and PEDOT:PSS can withstand this thermal budget, this process resolves the core issue of chemical incompatibility in traditional photolithography, providing a viable technical route for high-resolution patterning of PEDOT:PSS electrodes in organic electronic devices and flexible neural probes [84].

#### 6.1.3. Applications of PEDOT and Its Polymers in the Biomedical Field and Drug Delivery

**Figure 9:**
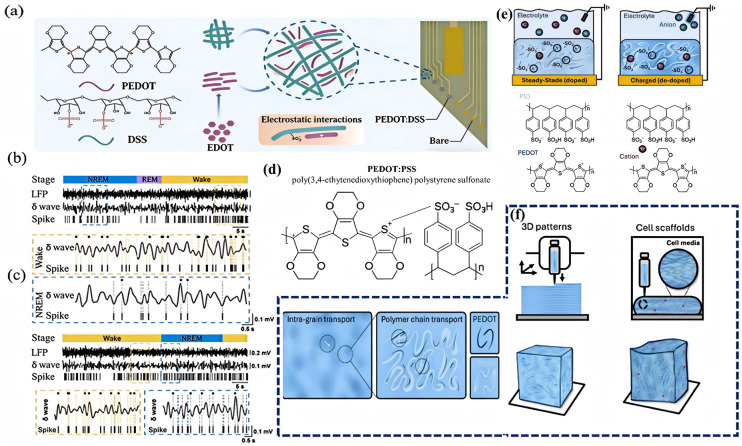
This figure illustrates the mechanism of the PEDOT: PSS electrodeposition process, whilst demonstrating through biological experimental data the benefits of this research for the application of PEDOT in the biological sciences. (**a**) Mechanism of PEDOT: DSS electrodeposition [105]. Reproduced with permission, Copyright 2025 *Biosensors & Bioelectronics*. (**b**) Discharge phases of representative LWENs relative to the delta wave, and the black dot above the dashed line indicates that the discharge was located close to the wave peak (scale bar, upper: 0.2 mV, lower: 0.1 mV) [113]. (**c**) Discharge phases of NRNs relative to the delta wave [118]. (**d**) Histograms of the delta phase of representative LWENs and fitted to the delta phase distributions by Von Mises (red solid line) (Rayleigh test; Wake: *p* = 5.74 × 10^−8^, *Z* = 16.65, *n* = 2540 spikes; NREM: *p* = 0.31, *Z* = 1.17, *n* = 128 spikes) [118]. (**e**) Delta wave phase-locking analysis of LWENs, ORNs and NRNs, with the red straight line representing the critical *p*-value (Rayleigh test, *p* = 0.05) [118]. (**f**) Delta phase distribution of representative NRNs is shown in 10° statistical intervals. The neuron exhibited phase-locking during wake (Rayleigh test; Wake: *p* = 7.58 × 10^−4^, *Z* = 7.18, *n* = 1989spikes; NREM: *p* = 0.11, *Z* = 2.24, *n* = 456 spikes). *p* < 0.05, *p* < 0.01, *p* < 0.001 and non-significance (n.s.) [118]. Reproduced with permission, Copyright 2026 *Journal of Materials Chemistry C*.

In the field of biomedical applications, to address the issues of signal attenuation [97,98,99] and interface failure [100,101,102] caused by immune reactions during long-term recording with implantable neural electrodes, researchers have achieved breakthroughs in electrode–nerve interface performance through the doping and structural design of PEDOT-based conductive polymers. Liu and colleagues addressed the bottleneck [103,104] caused by immune reactions during long-term recording with implantable neural electrodes, researchers have achieved breakthroughs in electrode-nerve interface performance through the doping and structural design of PEDOT-based conductive polymers. Liu and colleagues addressed the bottleneck [105],—by proposing the use of the anionic polysaccharide dextran sulfate (DSS) to replace PSS as the anionic template for PEDOT. They constructed a PEDOT:DSS-modified microelectrode array via rapid electrochemical deposition (Figure 9a) [106]. The introduction of DSS not only eliminated the acidic environment but also imparted a rougher surface topography (maximum roughness of 250 nm) to the coating, thereby reducing the electrode impedance at 1 kHz to 12.56 kΩ and increasing the charge storage capacity to 110.19 mC/cm^2^, which are 2.89 times and 2.89 times higher, respectively, than those of the PEDOT:PSS-modified electrode [105]. In vitro cell culture results showed that neural stem cell viability in the PEDOT:DSS group (82.46%) was significantly higher than that in the PEDOT:PSS group (49.03%), and the water contact angle decreased to 43.1°, indicating a significant improvement in hydrophilicity [105]. In a 7-day in vivo recording experiment in mouse brains, the PEDOT: DSS electrode exhibited a noise increase of only 23.89% and a signal-to-noise ratio (SNR) decrease of approximately 2.52%, whereas the PEDOT: PSS electrode showed a noise increase of 74.51% and an SNR decrease of 29.51%. The pore size and surface charge of the gel jointly determine the protein adsorption behavior and the long-term impedance evolution of the electrode. When the pore size is too small, proteins accumulate on the surface to form a protein corona, leading to a rapid increase in impedance; when the pore size is too large, proteins enter the pores and gradually block the ion channels, resulting in a delayed increase in impedance. The PEDOT main chain is positively charged in the doped state, which tends to adsorb negatively charged proteins to form a high-impedance layer. For PEDOT: DSS, the introduction of DSS reduces the surface charge density, leading to a more than 40% reduction in protein adsorption capacity—this explains why the noise increase (23.89%) of PEDOT: DSS in 7-day in vivo recordings is significantly lower than that of PEDOT: PSS (74.51%). Based on this high-performance electrode interface, this study achieved, for the first time, synchronous, high-signal-to-noise-ratio recordings of neurons in the lateral preoptic area and ventral pallidum of mice during the sleep–wake cycle, identified multiple state-dependent neuronal subtypes, and further discovered that the firing activity of some neurons during wakefulness exhibited significant phase locking with local field potential delta oscillations (0.5–4 Hz) (with peaks concentrated at 0°), whereas this phase locking disappeared during non-rapid eye movement (NREM) sleep, revealing a sleep–wake state-dependent mechanism of neural temporal coordination [107,108,109]. Kasavetov and his colleagues, however, focused on the mechanical brittleness of PEDOT: PSS films [110,111,112]. To address the technical limitations associated with the difficulty of increasing film thickness, a low-melting-point solvent formed by the combination of choline chloride (Sigma-Aldrich, St. Louis, MO, USA) and lactic acid (Sigma-Aldrich, St. Louis, MO, USA)was introduced into the PEDOT:PSS system, and GOPS cross-linking agent was added to prepare a PEDOT:PSS/DES eutectic gel [113]. Thanks to the introduction of DES, a single spin-coating step can yield films up to 800 nm thick (compared to only 117–199 nm for conventional PEDOT: PSS), reducing electrode resistance to 8.3 ± 1.6 kΩ and increasing charge storage capacity to 2.98 ± 0.19 mC/cm^2^ with a charge injection capacity of 0.49 ± 0.06 mC/cm^2^, representing an approximately 2.5-fold increase over conventional PEDOT: PSS. Acute rat sciatic nerve [114,115,116] implantation experiments confirmed that the PEDOT:PSS/DES electrode exhibited significantly lower impedance at 1 kHz, the signal-to-noise ratio was generally higher than that of PEDOT:PSS electrodes, and a reliable motor response of the tibialis anterior muscle could be reliably evoked at a lower stimulation current (approximately 0.4 V), whereas rigid gold electrodes showed no response under the same conditions and had a stimulation threshold as high as 2.0 V [27,117], indicating that this material has significant advantages in low-voltage neuromodulation [113] (Figure 9b–f).

#### 6.1.4. Applications of PEDOT and Its Polymers in Anti-Corrosion and Protective Coatings

**Figure 10:**
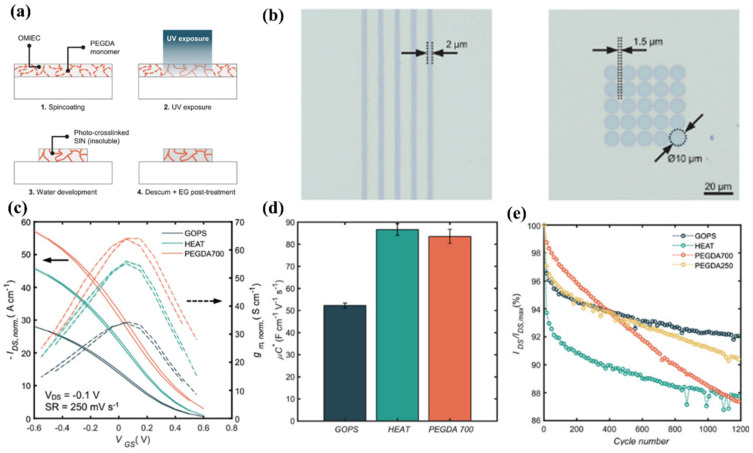
Comparison of Single-Layer PEDOT and Double-Layer OCDPD Composite Coatings. (**a**,**b**) shows PEDOT coatings on steel (**a**) and platinum (**b**) electrodes after 50 CV cycles, with a potential range of −0.5 to 1.8 V (SCE), a scan rate of 100 mV/s, and a temperature of 30 °C; (**c**–**e**) SEM images and EDX analysis of the steel surface: uncoated/1 h immersion [122]. (**c**), coated/1 h immersion [122]. (**d**), and coated/24 h immersion [122]. (**e**) in 0.05 M H_2_SO_4_ at 30 °C [122].

In the field of metal corrosion protection, research on conductive polymer coatings has long focused on the preparation, optimization, and performance characterization of single-layer PEDOT films. Ali and colleagues successfully electro-deposited dense and uniform PEDOT coatings onto steel electrodes using cyclic voltammetry and systematically investigated the effects of parameters such as scan rate, monomer concentration, deposition potential, and temperature on film quality [119]. The study found that the PEDOT coating obtained under optimized conditions (0.01 M EDOT, 100 mV·s^−1^ scan rate, −0.5 to 1.8 V potential range, 30 °C) exhibited a typical “cauliflower-like” microstructure (Figure 10a,b), after which the corrosion current density dropped to 4.47 × 10^−5^ mA·cm^−2^ following 24 h immersion in 0.1 M HCl solution—a reduction of approximately two orders of magnitude compared to bare steel, with a protection efficiency of 66%. Electrochemical impedance spectroscopy further revealed that, as immersion time increased, the charge transfer resistance of the PEDOT coating rose from 523 kΩ·cm^2^ at 1 h to 706 kΩ·cm^2^ at 24 h, indicating that it not only acts as a physical barrier to block corrosive media but also achieves synergistic protection by promoting the formation of an interfacial passivation layer (Figure 10c–e). The breakthrough of this design lies in the synergistic construction of multiple barriers: First, the OTMS-modified coating exhibits a water contact angle as high as 155.0° ± 0.7°, trapping a stable air layer at the solid–liquid interface based on the Cassie–Baxter model, thereby minimizing the contact area between the corrosive medium and the coating substrate. Second, the introduction of CeO_2_ continuously consumes oxygen diffusing to the interface through the Ce^4+^/Ce^3+^ redox reaction (2Ce^4+^ + O^2−^ → 2Ce^3+^ + ½O_2_), thereby suppressing the oxygen reduction reaction—a key step in corrosion—at its source. Third, the rigid framework of diatomaceous earth, combined with the crosslinked network of epoxy resin, forms a mechanically stable composite structure, ensuring that the superhydrophobic properties are maintained in complex environments.

Quantitative characterization results fully validate the superiority of this synergistic strategy: electrochemical testing shows that after 168 h of immersion in a 3.5 wt% NaCl solution, the surface resistivity f = 0.01 Hz of the OCDPD coating consistently remained three orders of magnitude higher (approximately 10^5^ Ω·cm^2^) than that of the bare magnesium alloy, with a corrosion inhibition efficiency as high as 99.11% [120]; in contrast, although the impedance modulus of the single-layer PD coating was slightly higher than that of the bare magnesium alloy, white NaCl particles began to aggregate as early as the ninth day of the salt spray test, indicating that its hydrophilic surface struggled to resist long-term chloride ion penetration. More critically, the OCDPD coating remained intact during the 21-day neutral salt spray test, whereas the single-layer superhydrophobic OCD coating exhibited minute black corrosion spots during the same test period, confirming that the bottom layer–top layer [120,121] configuration effectively blocks the vast majority of corrosive media from entering the system; even if trace amounts of media penetrate, the bottom layer can still provide secondary protection through electrochemical mechanisms. Through functional layered design, this study organically integrates the charge-shielding mechanism of PEDOT with the air-armor mechanism of the superhydrophobic coating, providing a novel material design paradigm for the long-term protection of active metals, such as magnesium alloys, in extreme corrosion environments.

In summary, conventional single-layer PEDOT coatings are difficult to serve long-term in chloride ion-containing environments due to their intrinsic hydrophilicity and limited barrier capability. In contrast, the functionally layered design—featuring an electroactive shielding underlayer and a superhydrophobic isolating top layer—achieves a corrosion inhibition efficiency exceeding 99% through functional decoupling. While this design paradigm is advanced, the fabrication process of the double-layer structure is complex and associated with high costs; furthermore, the batch stability during large-scale application and the self-healing capability after mechanical damage still require further optimization.

#### 6.1.5. Applications of PEDOT and Its Polymers in Energy Storage and Conversion

In the field of energy storage and conversion, PEDOT is widely used in supercapacitors, thermoelectric devices, and lithium-ion battery modification research due to its high electrical conductivity (reaching hundreds of S/cm in its intrinsic state), excellent electrochemical stability, and reversible redox activity. However, pure PEDOT systems have inherent limitations: in supercapacitors, due to restricted ion transport and volume expansion, pure PEDOT exhibits a specific capacitance of only approximately 120 F·g^−1^ and insufficient cycling stability [123,124]. In the thermoelectric field, although PEDOT:PSS has low thermal conductivity, its electrical conductivity and Seebeck coefficient are mutually limiting factors; conventional DMSO treatment can increase electrical conductivity by two orders of magnitude but results in a 30–40% decrease in the Seebeck coefficient [125,126]. In lithium-ion batteries, solution-coated PEDOT: PSS contains a large amount of insulating PSS (60–80 wt%) and exhibits poor adhesion to oxide substrates, making it difficult to provide stable protection under high voltages [127,128].

To address the performance bottlenecks of supercapacitors, Q et al. [129] combined multi-walled carbon nanotubes (MWCNTs) with PEDOT to construct a binder-free, self-supporting electrode. The three-dimensional conductive network of MWCNTs provides mechanical support while forming rapid electron transport pathways, and the elimination of traditional non-conductive binders eliminates interfacial resistance. Electrochemical quartz crystal microbalance (EQCM) analysis showed that the anion mobility in the composite film increased from 0.31 to 0.58, nearly doubling the charge compensation efficiency. This electrode exhibits a specific capacitance of 231 F·g^−1^ at 1 A·g^−1^, far exceeding that of pure PEDOT (120 F·g^−1^) and the MWCNTs/PVDF-HFP system (85 F·g^−1^), with a capacitance retention rate exceeding 90% after 5000 cycles. Multiple non-covalent interactions (hydrogen bonding, π–π stacking, fig of 275 F·g^−1^ at a current density of 1.0 A·g^−1^, an energy density of 34.04 Wh·kg^−1^, and a 180° peel strength of 4.2 N·cm^−1^) offer an innovative approach for flexible energy storage devices (Table 4).

## 7. Summary and Outlook

After more than three decades of development, research into PEDOT: PSS in the field of bioelectronics has evolved from preliminary explorations of conductive polymers into a multidimensional interdisciplinary field encompassing material synthesis, performance regulation, composite modification and bio-interface engineering. From the Bayer laboratories’ initial synthesis of PEDOT and the overcoming of its processing bottlenecks, to the systematic elucidation of the conductive mechanisms and doping mechanisms of PEDOT:PSS; from the establishment of a library of multi-component solution processing methods to the development of performance regulation strategies such as secondary doping, cross-linking stabilization and biofunctionalization; and from the validation of its in vitro cellular compatibility to the exploration of the feasibility of long-term in vivo implantation, these milestone achievements have laid the scientific foundation for PEDOT:PSS as one of the organic bioelectronic materials with the greatest application potential.

**Figure 11:**
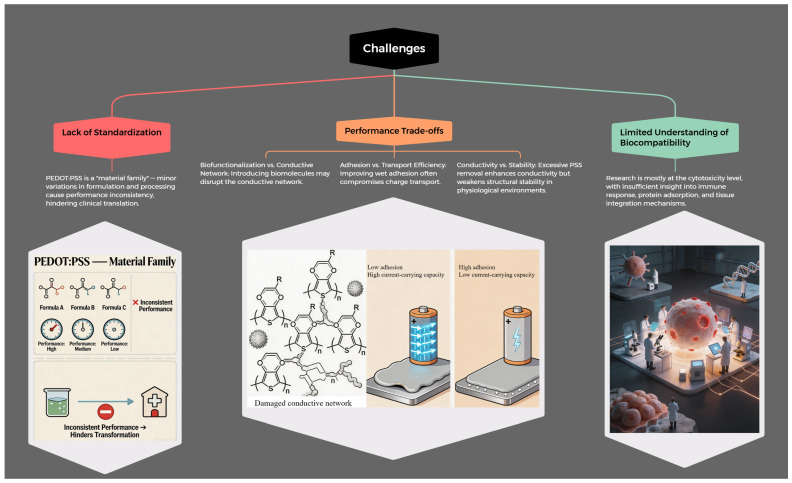
Issues identified in previous studies on PEDOT and challenges facing its future development.

Despite these achievements, the field still faces several challenges that require urgent resolution. First, the performance trade-off dilemma: There exists an inherent contradiction between high electrical conductivity and mechanical flexibility, as well as between charge storage capacity and long-term stability. Decoupling the coupled relationship between electrical conductivity and mechanical properties remains a core challenge. Second, the bottleneck of long-term stability: Most verification cycles only last from several days to several thousand cycles, which is far from meeting the 10-year service life required for clinical applications. Critical issues such as interface debonding, electrical conductivity degradation, and impedance increase induced by protein adsorption have not yet been systematically resolved. Third, minor variations in the formulation and processing of PEDOT: PSS lead to significant fluctuations in performance. The lack of unified reporting standards severely hinders horizontal comparisons and clinical translation of related technologies (Figure 11).

**Figure 12:**
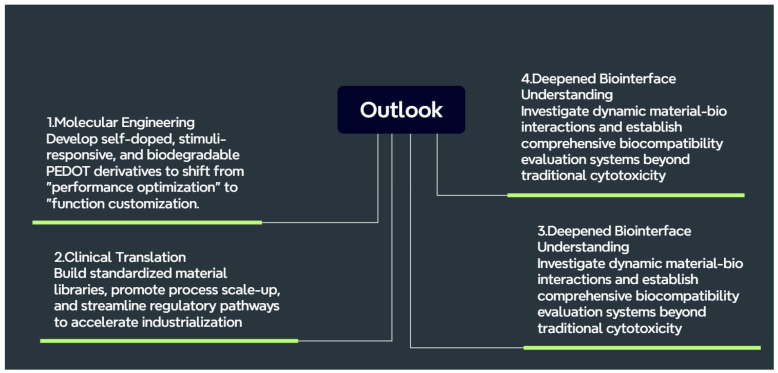
Outlook for the future development of PEDOT.

Future research into PEDOT:PSS will undergo a profound shift from “performance optimization” to ”functional customization”, advancing synergistically across three key areas: at the molecular engineering level, developing functional derivatives with properties such as self-doping, stimulus responsiveness and degradability to achieve precise control over the material’s intrinsic properties; at the bio interface level, moving beyond traditional cytotoxicity assessments to explore in depth the dynamic interaction mechanisms involved in immune responses, protein adsorption and tissue integration, thereby establishing a comprehensive multi-scale evaluation system; and at the clinical translation level, promoting the development of standardized material libraries and process scale-up, optimizing regulatory approval pathways, and accelerating practical applications in cutting-edge fields such as neural interfaces and wearable health monitoring. Synergistic breakthroughs across these three dimensions will inject strong momentum into the development of this classic conductive polymer in the new era (Figure 12).

## Figures and Tables

**Table 1:** Summary of electromechanical properties of PEDOT.

Property	Pure PEDOT	PEDOT: PSS	PEDOT-Based Composites	Ref.
Conductivity (S/cm)	~0.0075	10^−3^–10^3^ (max: 1.06 × 10^4^)	10^−3^–807 (varies by composite type)	[33]
**Carrier Mobility (cm^2^ V^−1^ s^−1^)**	Very low	1–10	Improved or tunable	[34]
**Carrier Concentration (cm** **^−^** **^3^** **)**	Extremely low	10^21^–10^22^	Increased or tunable	[35]
**Elastic Modulus**	Dense film: 1–5 GPa; Hydrogel: 2–10 MPa	Same as pure PEDOT	100 kPa^−5^ GPa	[36]
**Tensile Strength (MPa)**	10–50	8–40	0.81–50	[37]

**Table 2:** Comparison of synthetic methods for PEDOT.

Method	Key Features	Advantages	Limitations	Ref.
Solution-phase Synthesis	Oxidative polymerization in liquid; yields PEDOT powder or aqueous dispersion.	Mature process, low-cost, scalable batch production.	Poor morphology control, moderate intrinsic conductivity.	[43]
Vapor-phase/Interfacial Deposition	EDOT vapor polymerizes on oxidant-precoated substrates; supports patterned growth.	Ultra-high conductivity (>1000 S/cm), good patterning resolution.	Complex equipment, requires uniform oxidant deposition.	[42]
Electrochemical Polymerization	Anodic oxidation of EDOT on working electrode; polymerization and doping synchronize.	Precise control of film thickness/morphology.	Hard to scale up, restricted by electrode dimension.	[44]
Radiation Polymerization	High-energy radiation initiates polymerization via solvent-derived radicals.	No chemical initiators, tunable nanostructures.	Expensive apparatus, strict radiation safety rules.	[45]
Molecular Engineering Design	Pre-modified EDOT monomers or block copolymers.	Custom functions (biocompatibility, thermosensitivity, fluorescence).	Complex synthesis, low production yield.	[46]

**Table 3:** Comparison of the properties of PEDOT when combined with other substances.

Composite System	Performance Summary	Advantages	Disadvantages	Ref.
PEDOT + Conductive Polymers (PANI, PPy)	Conductivity: 10^−3^~10^3^ S/cm; Carrier mobility: 1~10 cm^2^ V^−1^ s^−1^; Carrier concentration: 10^21^~10^22^ cm^−3^; Elastic modulus: <1~5 GPa; Tensile strength: 8~40 MPa; Elongation at break: 15~60%; Flexibility: Stable under bending	High specific capacitance, continuous conductive network, improved flexibility	Prone to interfacial delamination, poor cycling stability, large performance fluctuation	[21]
PEDOT + Non-Conductive Polymers (PVA, PEO)	Conductivity: 10^−3^~10^3^ S/cm (slightly reduced); Carrier mobility: Slightly reduced; Carrier concentration: Slightly reduced; Elastic modulus: 0.36 GPa; Tensile strength: 38.7 MPa; Elongation at break: 142%; Flexibility: Excellent, bendable	High ductility, fast ion transport, good mechanical stability	Reduced conductivity, decreased electrochemical activity, trade-off between strength and conductivity	[22,23]
PEDOT + Metals/Metal Oxides	Conductivity: Up to 807 S/cm; Carrier mobility: Significantly improved; Carrier concentration: Significantly improved; Elastic modulus: 550 MPa; Tensile strength: 0.81 MPa; Elongation at break: >50%/473%; Flexibility: Stable under bending	Simultaneous enhancement of conductivity and flexibility, fast charge transport, improved strength	Easy particle agglomeration, poor biocompatibility, weak long-term stability	[25,46,59]
PEDOT + Inorganic Nanoparticles/Biomaterials	Conductivity: Tunable; Carrier mobility: Improved; Carrier concentration: Tunable; Elastic modulus: ~5 kPa; Tensile strength: Tissue-matching; Elongation at break: Tissue-matching; Flexibility: Biocompatible	Excellent biocompatibility, enhanced optoelectronic activity, nerve tissue-matching modulus	Weak conductivity, insufficient stability, high cost, complex preparation	[64,65]

**Table 4:** Comparison of the properties of PEDOT when combined with other substances.

Application Field	Core Advantages	Main Limitations	Ref.
**High-Performance Sensors & Data Transmission**	1. Ultra-high sensitivity, ultra-low detection limit and fast response; 2. Integrated capabilities of multimodal sensing, omnidirectional force detection and electromagnetic shielding; 3. Superior electrical conductivity and mechanical flexibility for flexible/wearable electronics	1. Inconsistent test conditions across studies hinder direct performance comparison; 2. Trade-off exists between ultra-high sensitivity and long-term cyclic stability; 3. Long-term reliability under complex practical environments remains to be verified	[87]
**Printing & Micropatterning**	1. Adaptable to photolithography, soft lithography and microcontact printing; 2. Fluorine-based etching enables precise depth control; SiN layer solves chemical incompatibility; 3. Enables high-resolution micropatterning for organic electronics and flexible neural probes	1. PEDOT: PSS films are susceptible to chemicals and moisture, limiting conventional photolithography; 2. Conventional lift-off method causes rough edge morphology and U-shaped cross-section; 3. Modified lithography with protective layers involves complicated procedures	[79,80,81,82,83]
**Biomedical Applications & Drug Delivery**	1. Reduced electrode–tissue impedance and enhanced charge storage/injection capability; 2. Improved biocompatibility and hydrophilicity, alleviating protein adsorption and immune response; 3. Enables high-SNR synchronous neuronal recording across sleep–wake cycles; 4. Superior low-voltage neuromodulation over rigid Au electrodes	1. Intrinsic brittleness of pristine PEDOT: PSS restricts thick-film fabrication; 2. Pore size and surface charge induce protein adsorption and long-term impedance drift; 3. Long-term in vivo biosafety and interfacial stability require further validation	[97,98,99]
**Anti-Corrosion Protective Coatings**	1. Dual protection via physical barrier and electrochemical passivation; 2. Functional bilayer design integrates superhydrophobic isolation and charge shielding; 3. Applicable to long-term corrosion protection of active metals such as Mg alloys	1. Monolayer PEDOT suffers from inherent hydrophilicity and limited barrier performance in chloride-containing media; 2. Bilayer composite coatings involve complex fabrication and high cost; 3. Scalable batch stability and self-healing ability need further optimization	[119]
**Energy Storage & Conversion**	1. High intrinsic conductivity, excellent electrochemical stability and reversible redox activity; 2. Hybridization with carbon nanotubes/polydopamine greatly enhances capacitance and cycling durability; 3. Versatile for supercapacitors, thermoelectrics and lithium-ion battery modification	1. Pristine PEDOT suffers from restricted ion transport and volume expansion; 2. Mutual restriction between electrical conductivity and Seebeck coefficient in thermoelectric systems; 3. Abundant insulating PSS in PEDOT: PSS causes poor substrate adhesion and inferior high-voltage stability	[123,124]

## Data Availability

Since the literature belongs to the review category, the data is derived from the cited literature and there are no other data sources.
